# Cell Surface Ion-Seq: Potassium Ion Monitoring in the Colorectal Cancer Cellular Microenvironment Based on Split G‑quadruplex Probe

**DOI:** 10.1021/acscentsci.5c01472

**Published:** 2025-12-05

**Authors:** Zhiyong Huang, Xuyang Shi, Yunben Yang, Wen Ma, Huimin Li, Zimin Jin, Yawei Feng, Cong Luo, Linfeng Zheng, Ziyan Du, Chang Liu, Chuanyu Liu, Yong Liu, Qin Wu, Longqi Liu, Ruizi Peng, Weihong Tan

**Affiliations:** † Department of Clinical Laboratory, Zhejiang Cancer Hospital, The Key Laboratory of Zhejiang Province for Aptamers and Theranostics, 631027 Hangzhou Institute of Medicine (HIM), Chinese Academy of Sciences, Hangzhou 310022, P. R. China; ‡ Molecular Science and Biomedicine Laboratory (MBL), State Key Laboratory of Chemo and Biosensing, College of Chemistry and Chemical Engineering, Aptamer Engineering Center of Hunan Province, 12569 Hunan University, Changsha, Hunan 410082, P. R. China; § 672740 BGI-Research, Hangzhou 310030, P. R. China; ∥ State Key Laboratory of Genome and Multi-omics Technologies, BGI-Research, Shenzhen 518083, P. R. China; ⊥ Shanxi Medical University-BGI Collaborative Center for Future Medicine, Shanxi Medical University, Taiyuan 030001, P. R. China; # Institute of Molecular Medicine (IMM), Renji Hospital, Shanghai Jiao Tong University School of Medicine, and College of Chemistry and Chemical Engineering, Shanghai Jiao Tong University, Shanghai 200240, P. R. China

## Abstract

Potassium ions (K^+^) are vital for biological systems and essential cellular functions, making their homeostasis monitoring crucial for investigating electrolyte imbalance symptoms. Antibody-based high-throughput single-cell sequencing enables simultaneous phenotype and genotype profiling in individual cells; however, its application to ion monitoring encounters inherent limitations. The subnanometer scale of K^+^ ions precludes antibody-based detection, creating a significant technical barrier for correlating ionic activity with cellular phenotypes at single-cell resolution. In this study, we report a cell surface biosensor for single-cell ion sequencing (Ion-seq) to monitor K^+^ homeostasis in clinical samples. The design utilizes split G-quadruplex (G4), where two guanine-rich DNA oligonucleotides cofold into four-stranded noncanonical secondary structures upon K^+^ binding. Specifically, a lipid-labeled capture probe anchors to the cell membrane. Upon drug-stimulated release of K^+^ from cells, the free sensing probe is captured, forming a complete G-quadruplex with the capture probe. Sequencing the sensing probe then enables monitoring of potassium homeostasis at single-cell resolution, allowing ionic phenotyping within the context of cellular heterogeneity and function. Moreover, this biosensor holds potential for broader bioapplications in analyzing other ions at the single-cell resolution, advancing disease diagnosis and personalized medicine.

## Graphical abstract

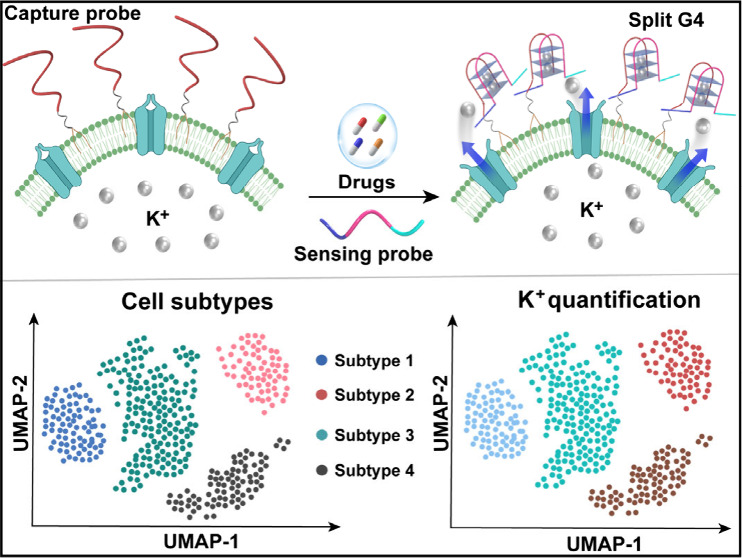

## Introduction

Potassium ions (K^+^) regulate crucial biological processes.^1,2^ Imbalances in potassium homeostasis are closely related to the onset and progression of electrolyte imbalance-associated diseases, including hypertension, cancer, and cardiovascular disease.^3,4^ For instance, hyperkalemia, characterized by elevated K^+^ levels in the blood, leads to severe conditions such as arrhythmias or even cardiac arrest. Conversely, hypokalemia, marked by low K^+^ levels, results in muscle weakness, fatigue, and paralysis in extreme cases.^5,6^ Moreover, during tumor development, increased extracellular K^+^ induces cancer cell necrosis, disrupting infiltrating T cell differentiation and severely compromising T cell-based tumor immunotherapy efficacy.^7,8^ Importantly, heterogeneous tumor microenvironment drives varied individual cell responses, significantly influencing tumor invasion, metastasis, and treatment effectiveness.^9,10^ Therefore, it is essential to consider the effects of cellular heterogeneity when monitoring K^+^ in clinical studies.^11^

Analytical methods have been developed for monitoring potassium homeostasis.^12^ Electroanalysis, for example, is based on ion-selective electrodes, and it has achieved a wide range of ion concentration detection with high spatiotemporal resolution.^13^ However, the technology suffers from a limited lifespan and low precision due to interference from other ions. Flame photometry provides sensitive, accurate, and cost-effective K^+^ detection, yet it requires tedious sample pretreatment, is susceptible to the interference from other metal ions, and has limited accuracy at high concentrations.^14^ Small-molecule-based fluorescent probes offer a noninvasive approach for ion monitoring, but are constrained by fluorescence crosstalk and photobleaching.^12,15,16^ Inductively coupled plasma mass spectrometry can analyze K^+^ in whole single cells, though sample preparation strategies have intrinsic limitations and may wash out K^+^.^17^ Critically, the output signals from these methods reflect the average status of clinical samples, obscuring inherent cellular heterogeneity. Recent advancements of high-throughput single-cell sequencing have revolutionized our ability to identify and monitor the phenotypes and dynamics of individual cells within complex populations.^18,19^ Technically, high-throughput single-cell sequencing for genotype or phenotypes has fundamentally relied on molecular probes, e.g., DNA-barcoded antibodies for proteomics studies.^20,21^ However, the lack of suitable probes for ions has hindered the analysis of ionic phenotypes containing K^+^ through high-throughput single-cell sequencing.

Interestingly, guanine-quadruplex (G4), a guanine-rich oligonucleotide, folds into four-stranded noncanonical secondary structures via Hoogsteen hydrogen bonds in the presence of K^+^.^22,23^ Biosensors exploiting the split form of G4 (split G4) have been developed for K^+^ detection, benefiting from easy modification, low cost, high specificity, high sensitivity, and high stability.^22,24^ Increasing the K^+^ concentration triggers assembly of two split G4 strands into a complete G4 structure. Leveraging this functional DNA oligonucleotide probe, we describe a technology, termed ion sequencing (Ion-seq), for K^+^ monitoring on the cell surface. This approach overcomes the aforementioned limitations and provides a true ion recognition probe. As a proof of concept, we achieved K^+^ monitoring in clinical samples via high-throughput single-cell sequencing (Figure 1). The working principle first involves a lipid-labeled capture probe (one split G4 strand) anchored on the cell membrane. Then, drugs were added to stimulate K^+^ efflux, enabling the free sensing probe (another split G4 strand) to form complete G4 structures with the capture probe. Correspondingly, an increased K^+^ release captures more sensing probes on the membrane. Finally, sequencing the sensing probe reveals heterogeneous K^+^ release in a colorectal cancer cellular microenvironment. This split G4-based Ion-seq technology thus provides a powerful tool for monitoring changes in ionic levels in single cells.

**Figure 1:**
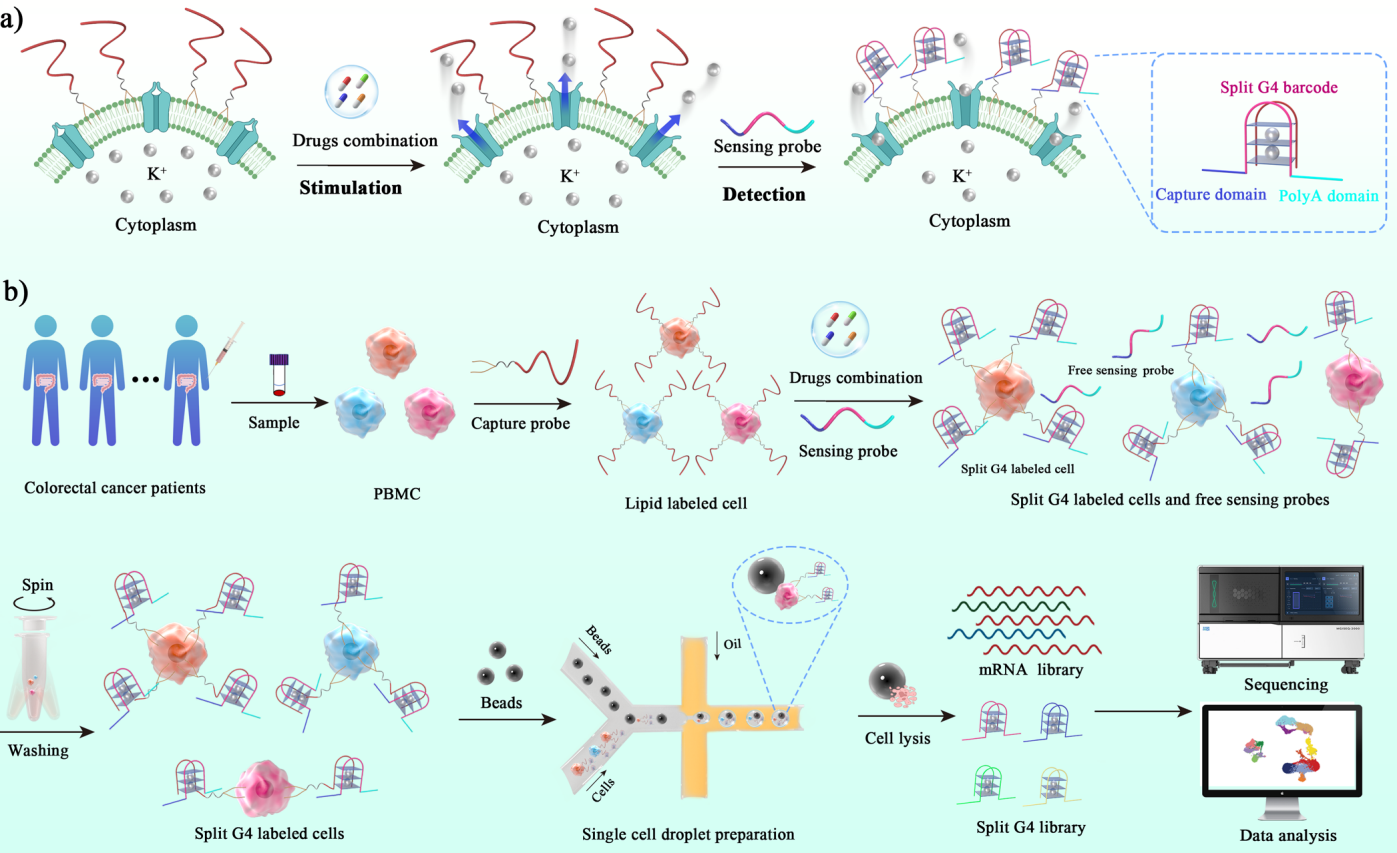
Potassium ion monitoring by Ion-seq in the colorectal cancer cellular microenvironment. (a) Schematic illustration of K^+^ monitoring in the cellular microenvironment based on the split G4 probe. Initially, the lipid-labeled capture probe was anchored on the cell membrane. After K^+^ efflux from cells by drug stimulation, the free sensing probe dimerized with the capture probe in the presence of K^+^, forming a G4 structure. By single-cell sequencing of the sensing probe, the changes of potassium concentration can be monitored at the single-cell level. (b) General process of K^+^ monitoring using Ion-seq. After the cells were isolated from colorectal cancer patients, the capture probe was anchored on the cell membrane. Then, the drugs were added, and the sensing probe was presented. Finally, Ion-seq was performed to show the ionic phenotype of isolated clinical cells from colorectal cancer patients.

## Results and Discussion

### Construction of Split G-Quadruplex Probe on the Cell Surface

To develop the split G4 probe for K^+^ monitoring, we initially prepared two split G4 strands. Based on the results from circular dichroism (CD, Figure S1) and fluorescence polarization (Figure S2), one G4 sequence (termed 93del, shown in Table S1)^22^ showed faster dynamics in response to the presence of K^+^. After respectively labeling fluorescent dyes Cy3 and Cy5 at the end of the two split strands, fluorescence resonance energy transfer (FRET) studies further verified that the 93del sequence exhibited higher sensitivity and selectivity compared to another group (Figure 2a and Figure S3). Therefore, the 93del sequence was used in the following studies. In order to adapt to single-cell sequencing, a poly adenine (PolyA) domain and a capture sequence domain were separately extended at the 5′- and 3′-ends of 93del, respectively, and termed the sensing probe. The CD result demonstrated that the extended domains had little influence on the formation of complete G4 in the presence of K^+^ (Figure S4). As the concentration of K^+^ increased, the FRET signal between Cy3-labeled 93del (one strand of split G4) and Cy5-labeled sensing probe gradually increased, indicating that K^+^ prompted the heterodimerization of this K^+^-dependent split G4 (Figure 2b). Additionally, the selected sequence pair showed high specificity for K^+^ among some common cations (Figure 2c).

**Figure 2:**
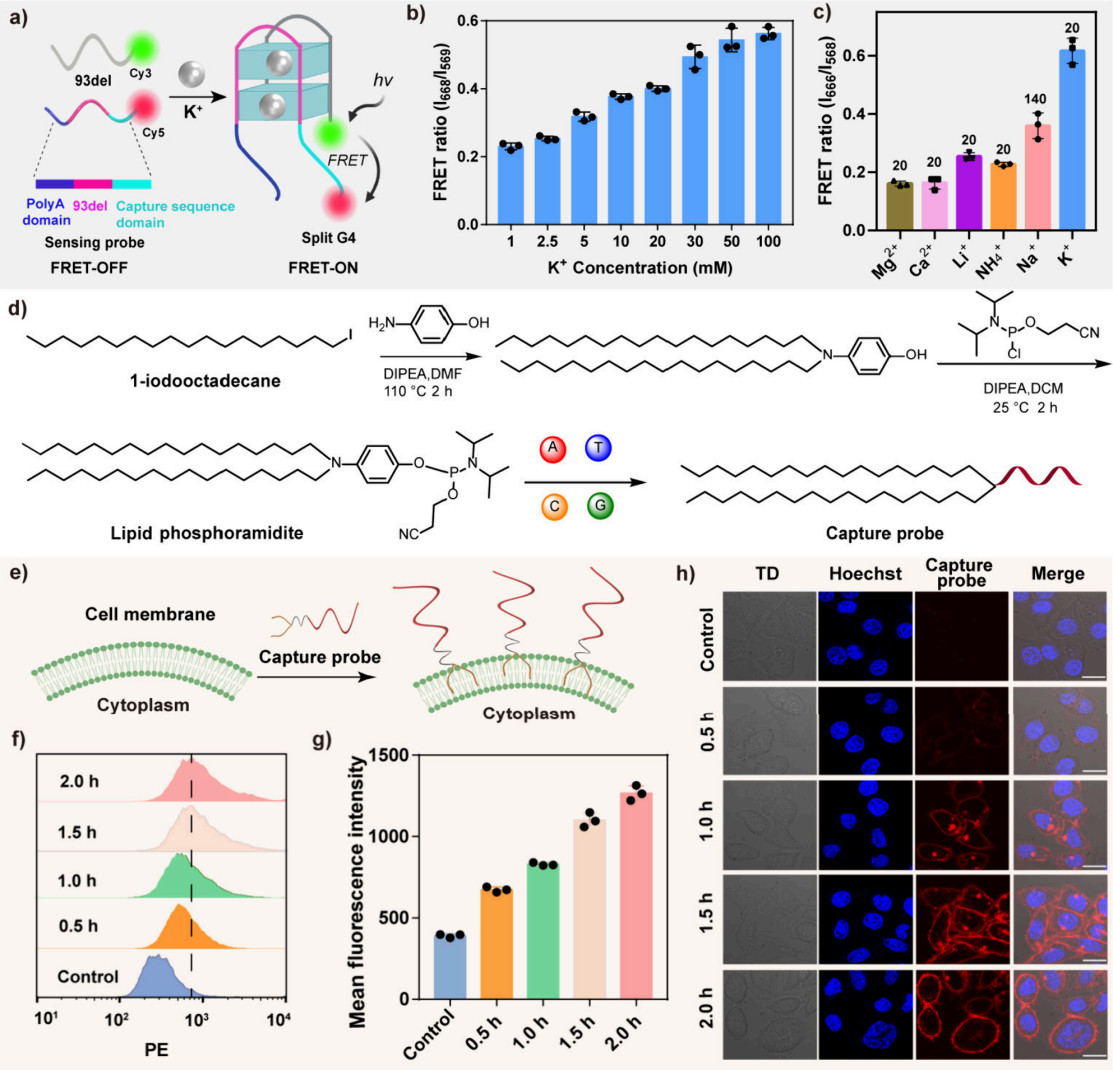
Development and validation of the split guanine-quadruplex probe on the cell surface. (a) K^+^-dependent formation of G4 by two split strands, including 93del (top) and sensing probe (bottom). Upon complete G4 formation, the FRET signal between Cy3 and Cy5, respectively labeled at the split strands, was observed. (b) Fluorescence analysis by FRET for different K^+^ concentrations that ranged from 0, 10, 20, 30, and 50 to 100 mM, demonstrating K^+^-dependent formation of complete split G4. (c) The high specificity of split G4 probes containing a 93del sequence with the different cations, including 20 mM K^+^, 140 mM Na^+^, 20 mM NH_4_^+^, 20 mM Li^+^, 20 mM Mg^2+^, and 20 mM Ca^2+^. (d) Synthetic route of conjugating the phosphoramidite lipid at the end of strand 93del (capture probe) and the construction of a capture probe for anchoring on the cell membrane. (e) Schematic illustration of on-membrane anchoring of the capture probe by hydrophobic lipid insertion. (f) Flow cytometry analysis of the capture probe incubated with LoVo cells for different times, including 0, 0.5, 1.0, 1.5, and 2.0 h. (g) Statistical analysis of fluorescence intensity corresponding to panel (f). Fluorescence intensity was measured by flow cytometry and expressed as the mean fluorescence intensity. All statistical data were collected from three independent experiments and presented as mean values ± SD. (h) CLSM imaging showed the anchoring of the capture probe on the LoVo cell membrane. Scale bars: 20 μm.

To obtain the cell-membrane-anchored capture probe, a lipid phosphoramidite was synthesized (Figures S5–S7). This hydrophobic lipid was covalently conjugated at the end of 93del through automatic solid-phase synthesis (Figure 2d), followed by purification with high-performance liquid chromatography and identification with mass spectrometry (Figure S8). Next, colorectal carcinoma LoVo cells were selected as the verification model. By incubating the prepared capture probe with LoVo cells, it was readily anchored onto the cell surface by hydrophobic lipid insertion (Figure 2e). The flow cytometry result showed a progressively increasing fluorescence intensity for the capture probe starting from 0 to 2 h, while little shift was observed for the 93del (free lipid-labeling control group), even after a 2 h incubation, confirming that the developed capture probe could effectively and specifically anchor onto the cell membrane (Figures 2f and 2g). Furthermore, confocal laser scanning microscopy (CLSM) imaging confirmed the positioning of the capture probe on the cell surface. As the incubation time increased, the fluorescence intensity on the cell surface increased, corroborating the flow cytometry result (Figure 2h).

### K^+^-Dependent Response with Split G4 on the Cell Surface

Since the sensing probe contains a 5′-polyA domain and a 3′-capture sequence domain, both extending from the separated ends of 93del, we investigated the feasibility of K^+^-driven complete G4 formation by the capture probe and the sensing probe on the cell surface. Initially, the Cy3-labeled capture probe was anchored to the cell surface by incubation. Subsequently, a Cy5-labeled 93del was incubated, as well as different concentrations of K^+^. The fluorescence study showed a gradually increasing Cy5 signal with the addition of K^+^, demonstrating the K^+^-dependent formation of complete G4 on the cell surface (Figure S9). Next, we determined K^+^-dependent formation of split G4 by a capture probe and sensing probe (Figure 3a). After the Cy3-labeled capture probe was anchored on the cell surface, higher concentrations of K^+^ were incrementally added and the free Cy5-labeled sensing probe was, indeed, captured, forming a stable complete split G4 structure. The Cy5 signal gradually increased as K^+^ concentration increased from 0 to 100 mM in flow cytometry (Figures 3b and 3c). The control group of 93del without lipid labeling could not anchor on the cell surface, and, hence, no fluorescence signal was observed in target channels. These results confirmed that increased structural stability of split G4 correlated with increased concentration of K^+^, thereby establishing the feasibility of monitoring K^+^ in the cellular microenvironment by Ion-seq.

**Figure 3:**
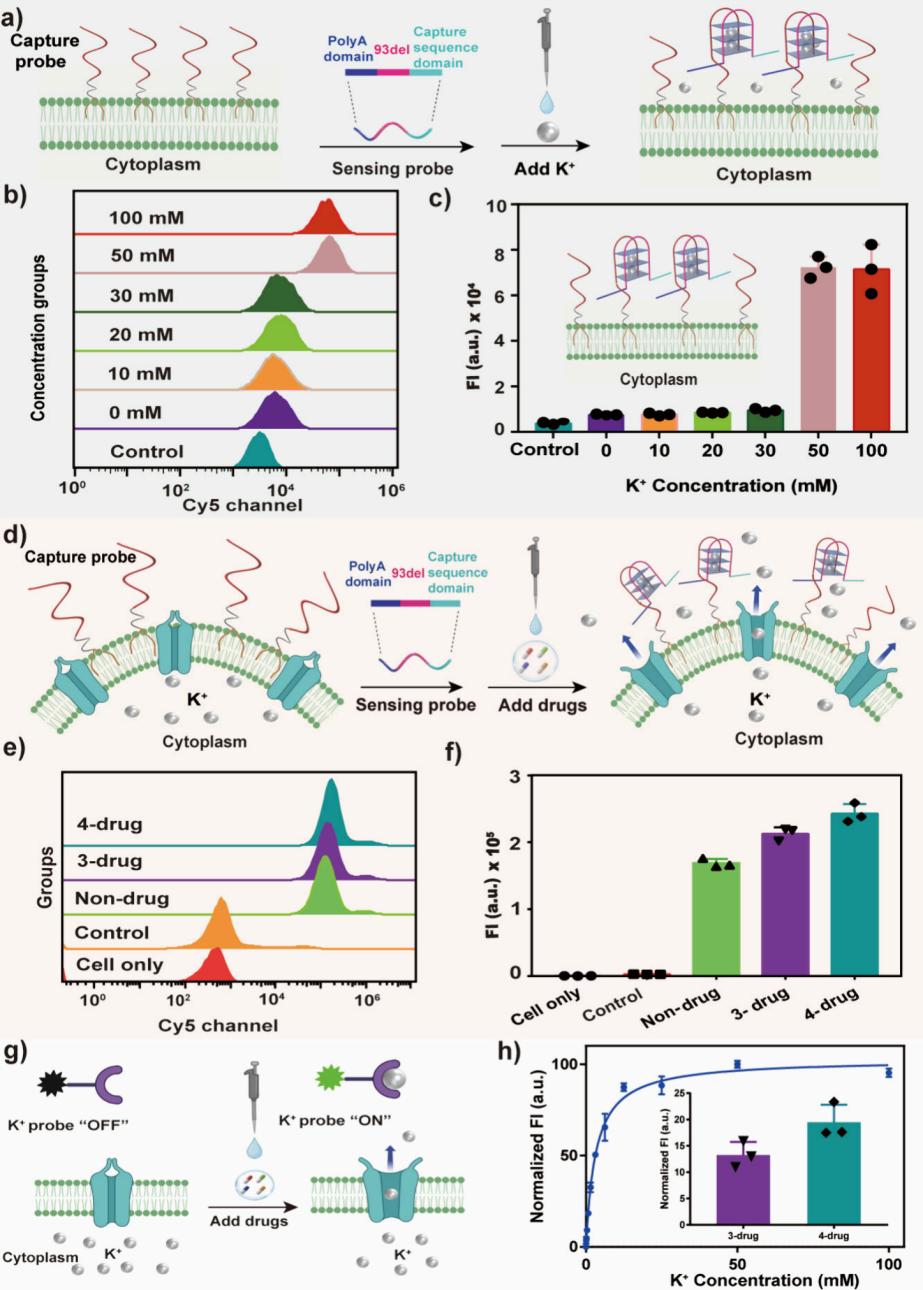
K^+^-dependent response by the split G4-based probe on the cell membrane. (a) Working principle of monitoring K^+^ by a split G4-based probe on the cell membrane. A Cy3-labeled capture probe was initially anchored on the cell membrane. As increasing concentrations of K^+^ were presented, the Cy5-labeled sensing probe was captured on the cell membrane, forming a G4 structure. (b) Flow cytometry analysis showed the K^+^-dependent response in the presence of different K^+^ concentrations, ranging from 0 to 100 mM. (c) Statistics of fluorescence intensity corresponding to panel (b). All statistical data were collected from three independent experiments and presented as mean values ± SD. (d) Fluorescence study of potassium ion efflux from living cells by drug stimulation: Schematic illustration of K^+^ sensing based on a split G4 probe on the cell membrane by drug stimulation. (e) Flow cytometry analysis showed the difference in Cy5 signal shift that resulted from treatment with the 3-drug group (Ampho B + Nig + Bum) and 4-drug group (Ampho B + Nig + Bum + Oua). (f) Statistics of fluorescence intensity corresponding to panel (e). All statistical data were collected from three independent experiments and presented as mean values ± SD. (g) Scheme of the commercial K^+^ probe that detected K^+^ efflux from living cells by drug stimulation. (h) Fluorescence titration curve of the commercial K^+^ probe under a series of K^+^ concentrations ranging from 1.6, 3.125, 6.25, 12.5, 25, and 50 to 100 mM and the determined K^+^ concentrations by 3- or 4-drug treatment. Excitation wavelength: 520 nm. Emission wavelength: 543 nm. All statistical data were collected from three independent experiments and presented as mean values ± SD.

After validating K^+^ monitoring on the cell surface by adding K^+^, we turned to the sensing of K^+^ efflux from living cells by drug stimulation (Figure 3d). In this study, four drugs were used, including amphotericin B (Ampho B) and nigericin (Nig), which stimulate K^+^ efflux from cells, as well as bumetanide (Bum) and ouabain (Oua), which inhibit K^+^ influx into cells (Figure S10).^25,26^ Initially, the Cy3-labeled capture probe was anchored on the LoVo cell membrane. Then, the added drugs costimulated K^+^ efflux from living cells, leading to the capture of Cy5-labeled 93del captured on the cell surface (Figure S11). To investigate K^+^ release by drug stimulation, a group containing 3 drugs (Ampho B + Nig + Bum) and another group containing 4 drugs (Ampho B + Nig + Bum + Oua) were chosen. After these groups were treated with respective drugs, the FRET signal in the 4-drug group was found to be higher than that in the 3-drug group, while little signal was shown in the nondrug treatment group, thus demonstrating effective K^+^ efflux sensing by drug stimulation (Figure S11). By employing a sensing probe, instead of the 93del strand, flow cytometry results also showed that the Cy5 signal in the 4-drug group was higher than that of the 3-drug group (Figures 3e and 3f and Figure S12), confirming the feasibility of K^+^ monitoring by drug stimulation for the sensing probe, which was subsequently used for high-throughput single-cell sequencing.

To achieve quantitative analysis of released K^+^ by drugs, a commercial small-molecule probe, IPG-4 TMA^+^, a cell-impermeable, fluorescent potassium (K^+^) indicator, was employed.^27^ Upon the addition of drugs, the K^+^ channels on the cell membrane were opened, expelling K^+^ from the cells. Such an expulsion was then sensed by IPG-4 TMA^+^ (Figure 3g). A fluorescence titration curve was obtained showing that this probe had suitable sensitivity on a millimolar scale of K^+^ and that it reached saturation at approximately 50 mM K^+^. Based on this standard curve, we quantitated released K^+^ by the 3-drug group and 4-drug group and found those values to be 0.6 and 0.9 mM, respectively (Figure 3h). The quantified K^+^ concentration, on average, indicated the effectiveness of different drug interventions in regulating K^+^ efflux and also explained the above results from flow cytometry (Figures 3e and 3f).

### Ion-seq Enables Ionic Phenotype Monitoring

After investigating K^+^ monitoring via fluorescence study, we performed Ion-seq to profile, in parallel, ion and transcriptome via high-throughput single-cell sequencing technology. As shown in Figure 1, the split G4 probe contains a PolyA domain, allowing barcode beads of the Ion-seq platform to directly capture the probe. The critical point of Ion-seq technology lies in sequencing a functional oligonucleotide (sensing probe) to reveal the target ion. Aptamers are highly selective functional oligonucleotides often used to analyze protein expression at the single-cell level by high-throughput single-cell sequencing.^28–30^ Since we wanted to broaden the idea of investigating generalized cellular phenotypes by sequencing the corresponding oligonucleotides, we turned to a DNA aptamer, sgc8c, targeting the cell membrane protein PTK7, as a model for validation (Figure S13).^31–33^ A scrambled strand was used as a control (Table S1). To optimize the aptamer capture efficiency of Ion-seq, the groups of only aptamers in the system (Test1), changing cell incubation conditions to the binding buffer in the system (Test2), changing cell lysis buffer in the Ion-seq system (Test3), and standard conditions as control (Test4) were compared using aptamer sgc8c with HCT-8 cell line. Analysis of single-cell sequencing data revealed the condition of the Test3 group reaching the highest capture efficiency (Figure 4a and Figure S14a). Subsequently, we employed aptamer sgc8c to evaluate the correlation between PTK7 mRNA and protein expression with HCT-8 cell line using the Ion-seq platform. Uniform manifold approximation and projection (UMAP) visualization revealed congruent spatial distributions of PTK7 mRNA and PTK7 aptamer binding across all cells (Figure 4b), demonstrating significant correlation between the aptamer’s protein targeting and transcript levels. Then, to assess whether the incubated oligonucleotide probes could affect the transcriptome, high-throughput single-cell sequencing was performed on the following groups in the colorectal cancer cell line HCT-8: free oligonucleotide-treated (binding buffer, group 1), Sgc8c aptamer-treated (group 2), and scramble strand-treated (group 3). After stringent quality control and filtering using multiple criteria, a mean of 3,000 genes and 4,500 unique molecular identifiers (UMI) were detected per cell, respectively (Figure S14b). Moreover, a high correlation (*r* ^2^ = 0.95, *p* < 2.2 e^–16^) of normalized gene expression was observed between the free oligonucleotide-treated group and the aptamer-treated group (Figure 4c). Additionally, the gene expressions shown in violin plots suggested that the distribution of genes with low- or high-abundance cell expression was comparable among the three groups (Figure S14c). Therefore, these results demonstrated that oligonucleotide probes did not affect gene expression. By high-throughput single-cell sequencing of the bound oligonucleotide probes on the cell membrane, the counts in box plots showed that those of the aptamer group was higher than those of the scramble group, demonstrating, in turn, that the membrane protein can also be specifically identified through sequencing the corresponding aptamer (Figure 4d). Therefore, these findings support our Ion-seq technology which calls for the sequencing of a functional oligonucleotide (sensing probe) to reveal the target ion. These results also show the universality of phenotype investigations by the dual strategy of sequencing oligonucleotide probes in combination with traditional single-cell RNA sequencing.

**Figure 4:**
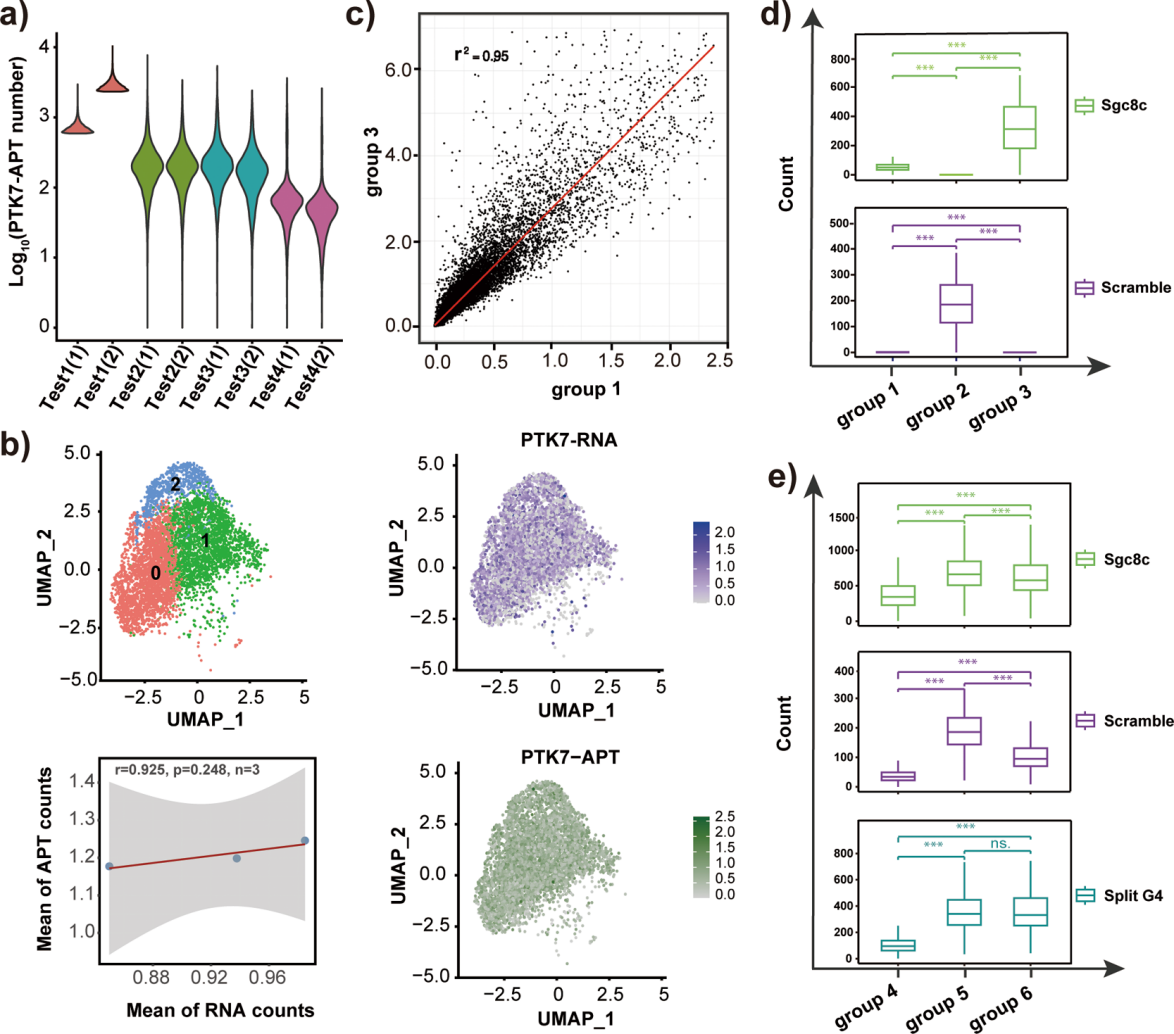
Validation of phenotype by sequencing a functional oligonucleotide probe at the single-cell level. (a) Violin plot showed the number of PTK7 aptamers captured per cell in various test groups. (b) Transcriptome-based clustering of HCT-8 cell line on the Ion-seq platform reveals distinct cell populations. The distribution abundance of PTK7 mRNA and aptamer were plotted on the UMAP. The mRNA was marked in purple and the corresponding aptamer was marked in green. Dot plots revealed the correlation between aptamer counts and mRNA expression levels of corresponding targets per cell, as measured by single-cell sequencing. (c) The dot plots showed the high correlation of gene expression between free oligonucleotide treated group and aptamer-treated group. (d) Box plots showed the aptamer count number for the three previously studied groups, demonstrating the feasibility of membrane protein detection by sequencing the corresponding oligonucleotide probe. (e) Box plots showed the sensing probe count number in split G4 among all groups, 3-drug-, 4-drug-, or non-drug-treated. All experimental data are expressed as the mean ± SD. Sequencing data were graphed and analyzed using R software. Statistical comparisons shown in the figure analyses were performed using a *t* test. Statistical significance: ***p < 0.001, ns, no significant difference.

To simultaneously profile ionic phenotype and transcriptome at the single-cell level, the previous drug combinations were employed to promote the release of K^+^ from LoVo cells, including the 3-drug group (Ampho B + Nig + Bum, group 5), the 4-drug group (Ampho B + Nig + Bum + Oua, group 6), and a non-drug-treated control group (group 4). Based on Ion-seq, the gene and UMI counts from these groups indicated high quality (Figure S15a). Furthermore, no significant differences were found in key gene expression among these groups (Figure S15b). According to the box plot, counts of the sensing probe in split G4 for the drug-treated groups were higher than those of the non-drug-treated group (Figure 4e), demonstrating that the developed Ion-seq technology could identify K^+^ efflux from living cells at single-cell scale.

### Potassium Ion Monitoring with Ion-seq in Clinical Samples

Next, we sought to test the efficacy of Ion-seq in profiling the dynamic changes of ions within peripheral blood mononuclear cell (PBMC) samples (Figure 5a). After collecting peripheral blood from colorectal cancer patients and isolating nucleated cells, we treated PBMCs with the previous drug combinations, including non-drug, 3-drug, and 4-drug. Each group was incubated with scramble, aptamer Sgc8c, and the developed split G4 probe. Then, we performed single-cell sequencing to identify ionic and transcriptomic changes, i.e., variations in gene expression patterns that occur in response to internal and external factors. After filtering out low-quality cells, we obtained transcriptome data sets from 46,022 cells with an average of 1,000 genes and 2,500 UMIs for each group (Figure S16a). To uncover immune cell populations in the PBMC sample, we performed unsupervised clustering and obtained 12 cell populations (Figure 5b). Immunocytes, such as T cells, B cells, monocytes, dendritic cells and erythrocytes, were identified based on the expression of classic cell type markers (Figure S16b). Using this approach, three populations were annotated as monocyte cells, including CD14^+^ monocytes (CD14, S100A12, LYZ), CD16^+^ monocytes (FCGR3A, CDKN1C, MS4A7), and intermediate monocytes (FCN1, CD14, FCGR3A);three populations were annotated as dendritic cells (CD1C, LYZ), erythrocytes (HBA1, HBD), and platelets (PPBP, PF4); four populations were annotated as T cells, including naive CD4^+^ T cells (CD3D, TCF7, LTB), NKT cells (NKG7, GZMB, GZMA), mucosal-associated invariant T cells (MAIT cells; CD3D, MAGI2), and cycling (proliferating) T cells (CD3D, MKI67, STMN1); two populations were annotated as B cells, including plasma cells (MZB1, IGHG1), and cycling B cells (MKI67, MZB1, STMN1) (Figure S16b).

**Figure 5:**
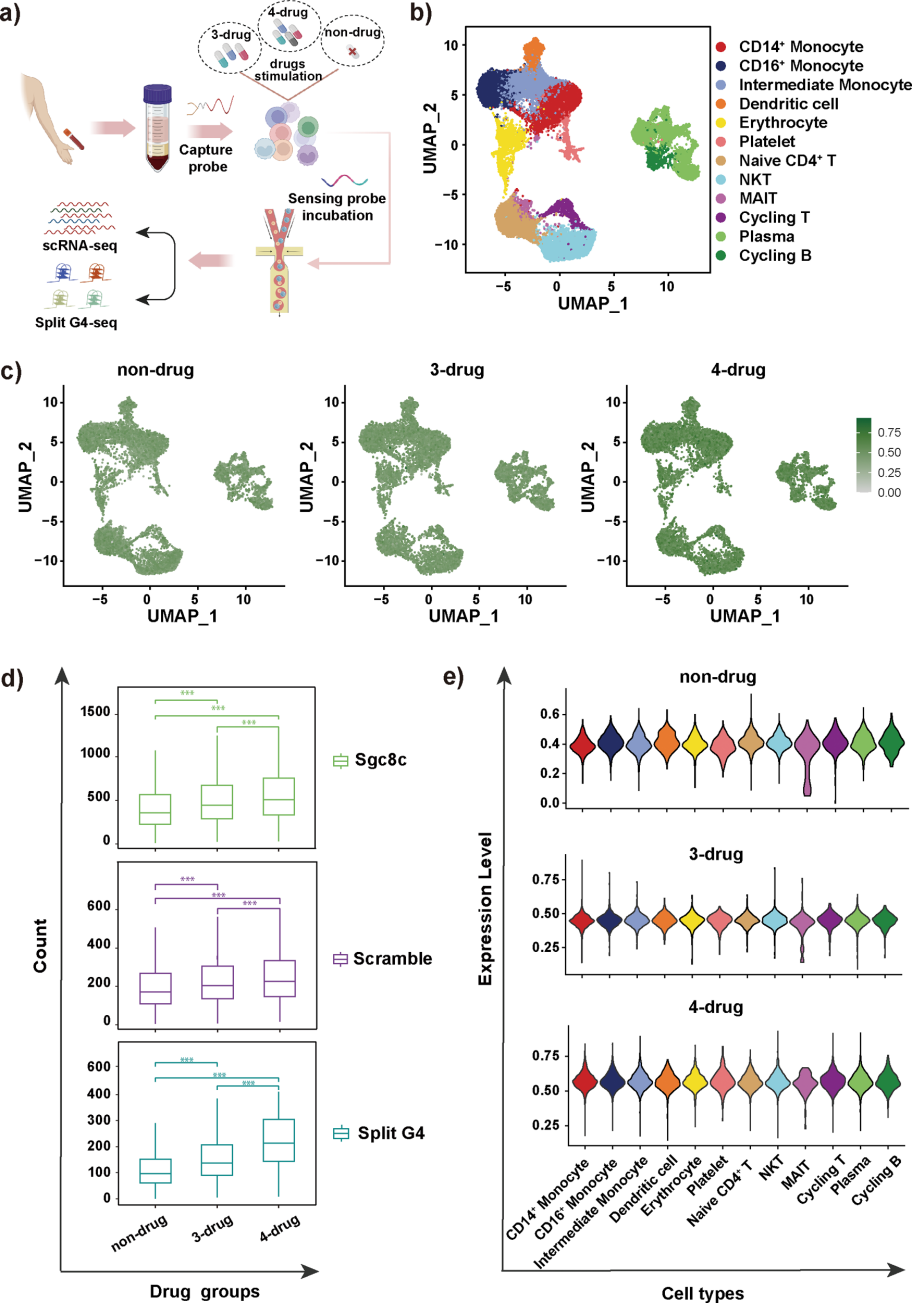
Potassium ion monitoring with Ion-seq in clinical samples. (a) Schematic illustrating the experimental design for profiling PBMC samples by Ion-seq. (b) Transcriptome-based clustering of single-cell expression profiles of PBMC reveals distinct cell populations. (c) UMAP visualization showing K^+^ levels based on split G4 aptamer binding in non-drug-treated (left), 3-drug treated (middle), and 4-drug treated groups (right). (d) Box plots show the count number for each of split G4, Sgc8c, and scramble among 3-drug-, 4-drug-, and non-drug-treated groups. (e) Violin plot showing K^+^ levels across distinct cell clusters in non-drug-treated, 3-drug treated, and 4-drug treated groups. All experimental data are expressed as the mean ± SD. Sequencing data were graphed and analyzed using R software. Statistical comparisons shown in the figure analyses were performed using a *t* test. Statistical significance: ***p < 0.001.

We then sought to determine whether split G4 probes were specifically distributed within particular subpopulations. To accomplish this, we performed a count of the sensing probes in split G4. Mapping split G4 probe on the UMAP plot revealed that drug treatment significantly enhanced the overall binding capacity, especially in 4-drug-treated groups (Figure 5c). Quantification confirmed higher sensing probe counts in the drug-treated groups compared to the non-drug-treated groups. Also, the number of sensing probes in the 4-drug-treated group was higher than that of the 3-drug group, demonstrating, again, that stimulation by different drugs had different effects, even on clinical cell samples (Figure 5d). Further analysis of the difference in K^+^ concentration levels across cell types in various groups demonstrated that, on the condition of drug-treatment, diverse cells exhibited a higher level of split G4 strand (Figure 5e). Drug treatment induces changes in the K^+^ concentration. To determine whether these K^+^ fluctuations impact the transcriptome, we analyzed differentially expressed genes (DEGs) across the three groups. The analysis revealed no significant DEGs, indicating that the altered K^+^ concentration does not induce significant transcriptomic alterations (Figure S16c). These results suggest that Ion-seq can identify changes in the K^+^ concentration within each cell type in complex colorectal cancer PBMC samples. Collectively, then, the data from both cell lines and colorectal cancer PBMCs confirmed that Ion-seq can effectively track dynamic K^+^ changes in living cells. This capability holds significant potential for advancing the understanding of signal transduction and metabolic processes and uncovering the pathological mechanisms underlying various diseases. Ultimately, this could lead to a new technology that could lead to the identification of advanced ion-based diagnostic and therapeutic biomarkers.

## Conclusion

Leveraging split G4 biosensors, we achieved potassium homeostasis monitoring on the cell surface within the colorectal cellular microenvironment using Ion-seq. The split G4 probe, as a functional oligonucleotide, allows a specific response to K^+^ concentration changes, providing a new molecular tool to reflect ionic fluctuations in cellular microenvironments with unprecedented precision. The synergy with advanced high-throughput single-cell sequencing enables Ion-seq to simultaneously profile transcriptomic and ionic phenotype changes at the single-cell resolution. Furthermore, sequencing these oligonucleotide probes demonstrates performance comparable to aptamer-based protein profiling. Consequently, monitoring the colorectal cancer microenvironment underscored the potential of Ion-seq for broader applications in clinical diseases and diverse sample types.

This study highlights several achievements: (1) A K^+^-dependent split G4 probe monitors ionic fluctuations with high specificity and high sensitivity, providing real-time insights into on-membrane microenvironments in clinical samples. (2) Integration with high-throughput single-cell sequencing resolves cellular phenotypic heterogeneity, minimizing the average signal from bulk solution. (3) Based on split G4, Ion-seq provides a probe enabling deeper understanding of ion homeostasis. (4) Ion-seq compares favorably to other oligonucleotide probes, such as aptamers, for profiling membrane proteins.

However, the Ion-seq method faces key limitations in quantification and extracellular potassium diffusion. Quantitatively, reliance on G-quadruplex formation often leads to saturating concentrations, resulting in nonlinearities between the signal and actual K^+^ concentration. Although the G4 probe folds quickly upon K^+^ release, the extracellular potassium diffusion fundamentally limits resolution, precluding the real-time observation of millisecond-scale ion events. And the formation speed of probes remains insufficient to capture ultrafast physiological fluctuations such as neuronal firing.^34^

Future development of oligonucleotide probes could advance clinical diagnosis, particularly for phenotype discovery in complex clinical samples. The Ion-seq for K^+^ monitoring using G-quadruplex probes opens avenues to expand the platform for monitoring Ca^2+^ and Mg^2+^, requiring the development of additional ion probes. Crucially, the modular design of Ion-seq enables multiplexed ion detection, permitting simultaneous tracking of K^+^, Ca^2+^, and Mg^2+^ dynamics within individual cells. Combining oligonucleotide probes with high-throughput single-cell sequencing will deepen the understanding at single-cell resolution, revealing new perspectives on physiological functions. This integration lays the foundation for advanced molecular probes to simultaneously profile ionomics, signal transduction, metabolomics, and transcriptomics. Moreover, the implications extend to therapeutics and personalized medicine, where single-cell phenotype identification could yield more targeted and effective therapeutic strategies.

## Supplementary Material



## Data Availability

The data related in this study are available in the CNGB Nucleotide Sequence Archive (CNSA: https://db.cngb.org/; accession number CNP0006677).

## Notes

DNA oligonucleotide information, HPLC program for purification of (Cy3-labeled) capture probe, clinical sample information, CD spectrum result, fluorescence polarization result, K^+^-driven FRET analysis, ^1^H NMR, ^13^C NMR and ^31^P NMR spectra, HPLC analysis and mass spectrometry analysis of the purity of capture probe, cytotoxicity study of capture probe and drug combinations, flow cytometry analysis and confocal imaging analysis, and single-cell data analysis (PDF)
