# Supplementary material for: Cell Surface Ion-Seq: Potassium Ion Monitoring in the Colorectal Cancer Cellular Microenvironment Based on Split G‑quadruplex Probe
Source: ACS Cent Sci. 2025 Dec 5;11(12):2375–84. doi: 10.1021/acscentsci.5c01472 (PMC12746146; doi:10.1021/acscentsci.5c01472)
Supplement: Supplementary file 1 [file oc5c01472_si_001.pdf]

Supporting Information

Cell Surface Ion-Seq: Potassium Ion Monitoring in the Colorectal Cancer Cellular Microenvironment Based on Split G-quadruplex Probe

Zhiyong Huang,[1,2,#] Xuyang Shi,[3,#] Yunben Yang,[1,#] Wen Ma,[3] Huimin Li,[1] Zimin Jin,[1] Yawei Feng,[1] Cong Luo,[1] Linfeng Zheng,[1] Ziyan Du,[1] Chang Liu,[3,4,5] Chuanyu Liu,[3,4,5] Yong Liu,[1] Qin Wu,[1] Longqi Liu,[3,4,5*] Ruizi Peng,[1,2,*] and Weihong Tan[1,2,6,*]

[1] Department of Clinical Laboratory, Zhejiang Cancer Hospital, Key Laboratory of Zhejiang Province for Aptamers and Theranostics, Hangzhou Institute of Medicine (HIM), Chinese Academy of Sciences, Hangzhou, Zhejiang 310022, P. R. China

[2] Molecular Science and Biomedicine Laboratory (MBL), State Key Laboratory of Chemo and Biosensing, College of Chemistry and Chemical Engineering, Aptamer Engineering Center of Hunan Province, Hunan University, Changsha, Hunan 410082, P. R. China

[3] BGI-Research, Hangzhou 310030, P. R. China

[4] State Key Laboratory of Genome and Multi-omics Technologies, BGI-Research, Shenzhen 518083, P. R. China

[5] Shanxi Medical University-BGI Collaborative Center for Future Medicine, Shanxi Medical University, Taiyuan 030001, P. R. China

[6] Institute of Molecular Medicine (IMM), Renji Hospital, Shanghai Jiao Tong University School of Medicine, and College of Chemistry and Chemical Engineering, Shanghai Jiao Tong University, Shanghai 200240, P. R. China

E-mail: tan@him.cas.cn; r_peng@hnu.edu.cn; liulongqi@genomics.cn

Content

1. Supplemental experimental details S3
    1.1 General reagents and materials S3
    1.2 Apparatus S3
2. Characterization of K^+-dependent split G4 probe S4
    2.1 Circular dichroism (CD) spectra analysis S4
    2.2 Fluorescence polarization analysis S4
    2.3 K^+-dependent response by FRET analysis S5
3. Synthesis route of capture probe S5
    3.1 Synthesis route of lipid phosphoramidite monomer S5
    3.2 Construction of capture probe S6
4. Cell experiments S7
    4.1 Cell culture S7
    4.2 General procedure for cell cytotoxicity tests S7
    4.3 Quantitative analysis of K^+ concentration by IPG-4 TMA^+ probe S7
    4.4 Flow cytometry analysis S8
    4.5 Confocal laser scanning microscopy (CLSM) imaging S9
5. Ion-seq S10
    5.1 The aptamer-based single-cell sequencing S10
    5.2 The split G4 based single-cell ion sequencing S11
    5.3 Clinical sample analysis S11
    5.4 Library construction and sequencing S11
    5.5 scRNA-seq data analysis S12
    5.6 Statistical analysis S12
    5.7 Data available S12
6. Supplemental figures S14
7. Supplementary tables S30
8. Ethics statement S33
9. References S34

1. Supplemental experimental details

1.1 General reagents and materials

All reaction reagents were purchased from Energy Chemical. Cell Counting Kit-8 was purchased from US Everbright Inc. (Suzhou, China). Hoechst 33342 (CAS: 23491-52-3) were purchased from Coolaber Science & Technology. 3,3'-dioctadecyloxacarbocyanine perchlorate (DiO, CAS: 34215-57-1) were purchased from Beyotime Biotechnology (Shanghai, China). CELLSAVING was purchased from New Cell & Molecular Biotech. HPLC-purified oligonucleotides (Table S1) were purchased from Shanghai Sangon Biotechnology Co., Ltd. Oligonucleotide stock solutions were prepared by dissolving DNA strands in ultrapure water (resistance = 18.2 MΩ/cm) and storing at -20 °C. The 3-drug group composition and concentration: 10 μM amphotericin B (Am), 10 μM ouabain octahydrate (Oua), and 10 μM bumetanide (Bum); the 4-drug group composition and concentration: 10 μM amphotericin B (Am), 10 μM nigericin (Nig), 10 μM ouabain octahydrate (Oua), and 10 μM bumetanide (Bum). For washing buffer, we used DPBS containing 4.5 g/L of glucose and 5 mM of $MgCl_2$, and for binding buffer, we used DPBS supplemented with 4.5 g/L of glucose, 5 mM of $MgCl_2$, 0.1 mg/mL of yeast tRNA, and 1 mg/mL of BSA. All chemicals were analytical grade and directly used without further purification. Sterile centrifuge tubes were purchased from Bioland Co., Ltd. (Hangzhou, China). 20-mm glass-bottom dishes and all plates for cell culture were purchased from NEST Biotechnology Co., Ltd. (Wuxi, China).

1.2 Apparatus

All solutions used in the experiments were prepared using ultrapure water obtained through Cascada™ Laboratory Water Purification Systems. Fluorescence kinetics measurements were performed with a Cary Bio-100 UV/Vis spectrometer (Prominence SPD-20A/20AV UV-Vis) for DNA quantification. Circular dichroism (CD) spectra were recorded on the Chirascan V100 Circular Dichroism Spectrometer (Applied Photophysics, UK). Confocal

images were captured by the A1 HD25 single-photon laser confocal microscope (NIKON, Japan). All ^{1}H, ^{13}C NMR, and ^{31}P NMR spectra were recorded on a Bruker 400 MHz at 20 °C. All ^{1}H, ^{13}C NMR, and ^{31}P NMR spectra were reported in parts per million (ppm) downfield of TMS and measured relative to the signals for TMS (0.00 ppm). Fluorescence polarization was recorded on a Spark 20M Microplate Reader (TECAN, Switzerland). The real-time fluorescence spectrum of the two samples was collected on a Cary Eclipse Fluorescence Spectrophotometer (Agilent Technologies, USA) at a measured frequency of 1 s.

2. Characterization of K^+-dependent split G4 probe

2.1 Circular dichroism (CD) spectra analysis

In this study, two candidates for split G4 probe were selected. Candidate 1 consisted of a 93del sequence (GGGGTGGGAGGAGGGT), while candidate 2 consisted of a G-quad sequence (TGAGGGAGGGG). In brief, 10.0 μM 93del were mixed with different concentrations of K^+ in 50 mM Tris-HCl buffer and incubated for 0.5 h, and then CD spectra were recorded on the Chirascan V100 Circular Dichroism Spectrometer (Applied Photophysics, UK).

Since the sensing probe contains 5'-polyA domain and 3'-capture sequence domains, both extending from the end of 93del, we recorded the CD spectra of 93del and sensing probe on the Chirascan V100 Circular Dichroism Spectrometer.

2.2 Fluorescence polarization analysis

To further confirm the optimal candidates, we conducted fluorescence polarization experiments for 93del and sensing probe. Different concentrations of K^+ were added to 5' Alexa Fluor 488-labeled 93del and incubated in a dark environment for 30 min with a final concentration of 150 nM. The solution was then added to 150 nM sensing probe and measured with the excitation wavelength of 488 nm and the emission wavelength of 525 nm (Tecan, Switzerland). Error bars show the standard deviation of three independent

experiments.

2.3 K^+-dependent response by FRET analysis

To investigate K^+-dependent response of the selected sequences, we carried out FRET analysis for Cy3-labeled 93del and Cy5-labeled 93del in the presence of different K^+ concentrations. Similarly, FRET analysis was performed for Cy3-labeled 93del and Cy5-labeled sensing probe. Fluorescence intensity was recorded on the Fluoromax-4 spectrofluorometer (HORIBA Jobin Yvon, Edison, NJ). For each experiment, 93del and KCl were mixed in a 90 μL HEPES buffer solution, the fluorescence intensity was recorded at 0.1 s intervals, and 10 μL of specific metal ions at a certain concentration were then added to form the split G4.

To test the specificity of split G4, the FRET ratio between Cy3-labeled 93del and Cy5-labeled 93del, was investigated. We investigated the FRET ratio between Cy3-labeled 93del and Cy5-labeled 93del in the presence of 20 mM KCl, 140 mM NaCl, 20 mM LiCl, 20 mM NH_4Cl, 20 mM $MgCl_2$, and 20 mM $CaCl_2$. Fluorescence intensity was recorded on the Fluoromax-4 spectrofluorometer (HORIBA Jobin Yvon, Edison, NJ).

3. Synthesis route of capture probe

3.1 Synthesis route of lipid phosphoramidite monomer

Synthesis of compound 2: Five grams (13.1 mmol) of 1-iodooctadecane, *N*, *N*-diisopropylethylamine (DIPEA, 3.40 g, 26.2 mmol), and 4-aminophenol (0.72 g, 6.6 mmol) were dissolved in anhydrous dimethylformamide (DMF, 60 mL), and the mixture was stirred at 110 °C. Thin-layer chromatography (TLC) was used to monitor the reaction. When the reaction was completed in about two hours, the mixture was allowed to cool to room temperature, followed by washing with saturated $NaHCO_3$ (200 mL) and extraction three times with 150 mL dichloromethane. The organic layer was collected and dried over anhydrous Na_2SO_4. The solvent was removed by rotary evaporator, followed by separation through a flash chromatographic column (PE: EA =30:1). Finally, 2.5 g of

compound 2 were obtained as a light brown solid product (62% yield).

Synthesis of lipid phosphoramidite 3. 0.7 g compound 2 (1.1 mmol) were dissolved in 20 mL anhydrous dichloromethane under N_2 atmosphere, and 0.3 g DIPEA (2.3 mmol) were added. The bottle was placed in an ice bath, and 0.4 g 2-Cyanoethyl *N*, *N*-diisopropylchlorophosphoramidite (1.7 mmol) were added dropwise. Then, the reaction was allowed to warm to room temperature and stirred for an additional 1 hour. Afterwards, the solvent was removed by rotary evaporator, followed by separation through a flash chromatographic column. Finally, lipid phosphoramidite 3 was obtained (0.7 g, 75% yield) as a colorless solid.

3.2 Construction of capture probe

Automatic solid-state synthesis of capture probe. The capture probe was directly purified by reversed-phase HPLC using a C4 reversed phase HPLC column. DNA synthesis reagents were purchased from Glen Research (Sterling, VA, USA). Oligonucleotide sequences were synthesized on a PolyGen synthesizer by the solid-state method, following the protocol of a previous report[1]. Lipid phosphoramidite dissolved in CH_2Cl_2 at the final concentration of 0.1 M was coupled onto the 5' ends of oligonucleotides as a final 'base' and subjected to coupling on a DNA synthesizer for 600 s. Afterwards, the synthesized oligonucleotides were deprotected, cleaved from the CPG, and precipitated in ethanol at -20 °C overnight. After centrifugation, the precipitate was dissolved in 0.1 M TEAA and purified by high-performance liquid chromatography (HPLC). Finally, the concentration of these oligonucleotides was calculated based on the absorbance at 260 nm with a UV-vis spectrometer (Cary Bio-300) according to the Lambert-Beer law.

Purification of capture probe by HPLC. The capture probe was purified by HPLC under the following conditions: C4 column, 25 °C; flow rate: 3.0 mL/min; eluent A: 0.1 M TEAA (pH 7.0); eluent B: acetonitrile; linear gradient, 5-65% B in 30 min; detection wavelength: 260 nm. The elution procedure is described in Table S2.

4. Cell experiments

4.1 Cell culture

LoVo and HCT-8 cells were purchased from the American Type Culture Collection and cultured in complete culture medium (RPMI-1640 (BasalMedia Technologies, L210KJ) supplemented with 10% FBS (Bio-Channel, BC-SE-FBS01) and 100 U/mL PS (MeilunBio, MA0110)) at 37 °C in a 5% CO_2 atmosphere.

4.2 General procedure for cell cytotoxicity tests

To evaluate the cytotoxicity of the capture probe, cells were seeded at a density of 30,000 cells per well in a 96-well plate and cultured at 37 °C in a 5% CO_2 atmosphere for 12 h. Then, different concentrations of the capture probe were added to wells of the 96-well plate and incubated with LoVo cells for 2 h at 37 °C. After 2 h incubation, the 1640 medium was removed, and cells were treated with fresh medium containing 10 μL of CCK8 for 1 h at 37 °C. Finally, absorbance of the medium at 450 nm was recorded with a microplate reader (Tecan, Switzerland).

Drugs stimulate apoptosis by promoting K^+ efflux, therefore, to ensure that cell viability would meet the requirements for single-cell sequencing, we also explored cell cytotoxicity by 3-drug (Ampho B + Nig + Bum) or 4-drug (Ampho B + Nig + Bum + Oua) treatments. First, LoVo cells were seeded at a density of 10,000 cells per well in a 96-well plate and cultured at 37 °C in a 5% CO_2 atmosphere for 12 h. Then, the 3- or 4-drug were added to wells of a 96-well plate and incubated with LoVo cells for 2 h at 37 °C. After 2 h incubation, the 1640 medium was removed, and cells were treated with fresh medium containing 10 μL of CCK8 for 1 h at 37 °C. Finally, absorbance of the medium at 450 nm was recorded with a microplate reader.

4.3 Quantitative analysis of K^+ concentration by IPG-4 TMA^+ probe

We used a commercial potassium probe (IPG-4 TMA^+) to quantify potassium ion efflux from cells. First, we investigated the saturation binding curve of K^+. LoVo cells were seeded at a density of $1.0×10^6$ cells per well in a 96-well plate

and cultured at 37 °C in a 5% CO_2 atmosphere for 12 h, followed by staining with IPG-4 TMA^+ (ION Biosciences, 3023F). Finally, different concentrations of K^+ (0, 1.6, 3.125, 6.25, 12.5, 25, 50, and 100 mM) were added to the LoVo cells, which were stained with 3 μM IPG-4 TMA^+.

To quantify K^+ efflux from cells, the 3- or 4-drug groups were added to wells of a 96-well plate and incubated with LoVo cells for 2 h at 37 °C. After 2 h incubation, LoVo cells were stained with 3 μM IPG-4 TMA^+. Fluorescence intensity of each sample was measured at predetermined time points using a fluorescence spectrometer (IPG-4 TMA^+, λ_{ex} = 520 nm, λ_{em} = 545 nm).

4.4 Flow cytometry analysis

To carry out the cell membrane anchoring assay, LoVo cells were seeded at a density of 1.0×10^6 cells per well in a 96-well plate, cultured at 37 °C in a 5% CO_2 atmosphere for 12 h, and then washed 3 times with DPBS, followed by incubation with 100 nM Cy3-labeled capture probe for 1 h in RPMI-1640 culture medium. After 1 h incubation, the cells were digested with trypsin-EDTA (0.25%) and washed another 3 times to remove the excess probes. Samples were then analyzed on a BD FACSVerse™ flow cytometer. Flow cytometry was performed on a FACScan cytometer by counting 10,000 events.

The formation of split G4 on the cell membrane by adding K^+ was investigated next. First, 1.0×10^6 LoVo cells were incubated with 100 nM Cy3-labeled capture probe at room temperature for 60 min. After removal of free Cy3-labeled capture probe, the modified cells were incubated with 200 nM Cy5-labeled 93del or sensing probe and different concentrations of K^+ (0,10, 20, 30, 50, and 100 mM) for another 30 min. To get the maximum unquenched fluorescence signal, a control experiment was conducted by replacing the Cy3-labeled capture probe with Cy3-labeled 93del in RPMI-1640 culture medium. After removal of free Cy5- labeled 93del or sensing probe, the resultant cells were suspended in 1.0 mL DPBS for flow cytometric analysis on a CytoFLEX LX Flow Cytometer (10000 cells collected, Beckman Coulter). Since oligonucleotides are negatively charged hydrophilic macromolecules and cannot freely permeate the

lipophilic cell membrane, Cy3-labeled 93del could only hybridize Cy5-labeled 93del, or sensing probe, on the extracellular side of the cell membrane. In other words, the split G4 should mainly localize on the outer leaflet of the cell membrane. Cells were washed with washing buffer before and after incubation.

Finally, the formation of split G4 on the cell membrane by adding different combinations of drugs to release K^+ was investigated. 1.0×10^6 LoVo cells were incubated with 100 nM Cy3-labeled capture probe at room temperature for 60 min. After removal of free Cy3-labeled capture probe, the modified cells were incubated with 200 nM Cy5-labeled 93del, or sensing probe, and the 3-drug group or 4-drug group for another 30 min. To get the maximum unquenched fluorescence signal, a control experiment was conducted by replacing the Cy3-labeled capture probe with Cy3-labeled 93del in the RPMI-1640 culture medium. After removal of free Cy5-labeled 93del. or sensing probe, the resultant cells were suspended in 1.0 mL DPBS for flow cytometric analysis on a CytoFLEX LX Flow Cytometer (10,000 cells collected, Beckman Coulter).

4.5 Confocal laser scanning microscopy (CLSM) imaging

The cell membrane anchoring assay was then investigated by CLSM imaging**.** LoVo cells were placed in a 20-mm cell culture dish, incubated overnight for adherence, and grown to 80-90% confluence before the experiment. LoVo cells were washed with DPBS twice, incubated with 100 nM Cy3-labeled capture probe for different times at 37 °C, washed three times with DPBS, and visualized by fluorescence imaging performed on the confocal laser scanning microscope equipped with 100x oil objective. Hoechst 33342 was used to stain the cell nucleus, and DiO was used to stain the cell membrane. The Cy3-labeled capture probe could also be inserted into the cell membrane so that DiO and Cy3-labeled capture probe could colocalize.

The formation of split G4 on the cell membrane by adding K^+ was also investigated. First, 1.0×10^6 LoVo cells were incubated with 100 nM Cy3-labeled lipid 93del at room temperature for 60 min. After removal of free Cy3-labeled capture probe, the modified cells were incubated with 200 nM Cy5-labeled

93del, and different concentrations of K^+ (0,10, 20, 30, 50, and 100 mM) for another 30 min. To get the maximum unquenched fluorescence signal, a control experiment was conducted by replacing the Cy3-labeled lipid 93del with Cy3-labeled capture probe in the RPMI-1640 culture medium. After removal of free Cy5-labeled 93del, the resultant cells were imaged on the confocal laser scanning microscope equipped with 100x oil objective. Hoechst 33342 was used to stain the cell nucleus, and DiO was used to stain the cell membrane. The Cy3-labeled capture probe could also be inserted into the cell membrane so that DiO and Cy3-labeled capture probe could colocalize.

The formation of split G4 on the cell membrane by adding different combinations of drugs was then investigated. First, 1.0×10^6 LoVo cells were incubated with 100 nM Cy3-labeled capture probe at room temperature for 60 min. After removal of free Cy3-labeled capture probe, the modified cells were incubated with 200 nM Cy5-labeled 93del, and different combinations of drugs for another 30 min. To get the maximum unquenched fluorescence signal, a control experiment was conducted with Cy3-labeled 93del in the RPMI-1640 culture medium. After removal of free Cy5-labeled 93del, the resultant cells were imaged on the confocal laser scanning microscope equipped with 100x oil objective. Hoechst 33342 was used to stain the cell nucleus, and DiO was used to stain the cell membrane. The Cy3-labeled capture probe could also be inserted into the cell membrane so that DiO and Cy3-labeled capture probe could colocalize.

5. Ion-seq

5.1 Aptamer-based single-cell sequencing

To verify the feasibility of single-cell sequencing based on oligonucleotide probes, we selected aptamer Sgc8c as a model. First, 1.0×10^6 cells were washed with washing buffer and then incubated with 200 nM Sgc8c in 400 μL binding buffer at 4℃ for 60 min. After 1 h incubation, the cells were washed 3 times with 500 μL washing buffer, and then cell viability was determined using

a cell counting plate. Finally, 22,000 cells were taken for sample preparation for subsequent single-cell sequencing.

5.2 Split G4-based single-cell ion sequencing

To simultaneously profile ions and transcriptomics at the single-cell level using this system, split G4 was selected as a model. First, 1.0×10^{6} cells were washed 3 times with washing buffer and then incubated with 200 nM capture probe in 400 μL binding buffer at room temperature for 60 min. After removal of free capture probe, the modified cells were incubated with 400 nM sensing probe and different combinations of drugs for another 30 min. LoVo cells were washed 3 times with washing buffer, followed by determining cell viability using a cell counting plate. Finally, 22,000 LoVo cells were taken for sample preparation for subsequent single-cell sequencing.

5.3 Clinical sample analysis

Peripheral blood samples were collected from colorectal cancer patients (Table S3).10 mL of peripheral blood from each donor were collected in an EDTA-anticoagulant tube. The sample was transferred to the laboratory within 1 h of collection. Written informed consent was obtained from the participants.

To isolate PBMCs, 20 mL of Ficoll-Paque Plus Buffer were added to a clean 50 mL tube. Then, 10 mL of peripheral blood, diluted with an equal volume of DPBS, were carefully layered on top of the Ficoll buffer. This mixture was centrifuged at 800 g at 25°C for 20 minutes. The buffy coat was then carefully transferred to a new tube and diluted with DPBS to a total volume of 20 mL. The sample was centrifuged again at 600 g for 6 min, the buffer was discarded, and the cell pellet was resuspended in 3 mL DPBS.

5.4 Library construction and sequencing

The DNBelab C Series Single-Cell Library Prep Set (MGI, cat.1000021082) was used as previously described to construct scRNA-seq libraries. Briefly, single-cell suspensions of PBMCs were used for droplet generation, emulsion breakage, bead collection, reverse transcription, and cDNA amplification to generate barcoded libraries. The sequencing libraries were quantified using the

Qubit™ ssDNA Assay Kit (Thermo Fisher Scientific). DNA nanoballs were then loaded into the patterned Nanoarrays and sequenced on an ultra-high-throughput DIPSEQ T1 sequencer. The read structure was paired-end with Read 1 that covered 30 bases inclusive of 10-bp cell barcode 1, 10-bp cell barcode 2 and 10-bp unique molecular identifier (UMI), Read 2 that contained 100 bases of transcript sequence and Read 3 that contained 10-bp sample index.

5.5 scRNA-seq data analysis

High-quality scRNA sequencing data with valid barcodes were aligned to the human reference genome (GRCh38) using STAR, and the unique molecular identifier (UMI) count matrix was generated using PISA (version 1.10.2) (https://github.com/shiquan/PISA). Each gene expression matrix from the PBMC datasets was imported into the R package Seurat (version 4.0.1) for clustering analysis.

First, cells with mitochondrial gene counts greater than 10% were excluded, as were cells expressing fewer than 400 or more than 4,500 genes. DoubletFinder (version 2.0.3) was employed to remove doublets, assuming a 5% doublet formation rate. The filtered data were then normalized and scaled using the “NormalizeData” and “ScaleData” functions with default parameters, respectively. The top 2000 highly variable genes for each library were used for further processing. Next, all datasets were integrated using the “FindIntegrationAnchors” and “IntegrateData” functions in Seurat. Finally, we conducted dimension reduction for the scaled merged dataset using PCA analysis. The first 30 principal components were used to construct the K-nearest neighbor graph (k-NNG) through the “FindNeighbors” function, and cell clusters were assigned using the “FindClusters” function. Visualization was performed using UMAP. Each cell type was annotated based on reported marker genes.

5.6 Statistical analysis

All experimental data are expressed as the mean ± standard deviation, and the

t-test was performed for all two-group comparisons. Sequencing data were graphed and analyzed using R software. Statistical significance was set at $P < 0.05$.

5.7 Data available

The data related in this study are available in the CNGB Nucleotide Sequence Archive (CNSA: https://db.cngb.org/; accession number CNP0006677) [4,5].

6. Supplemental figures

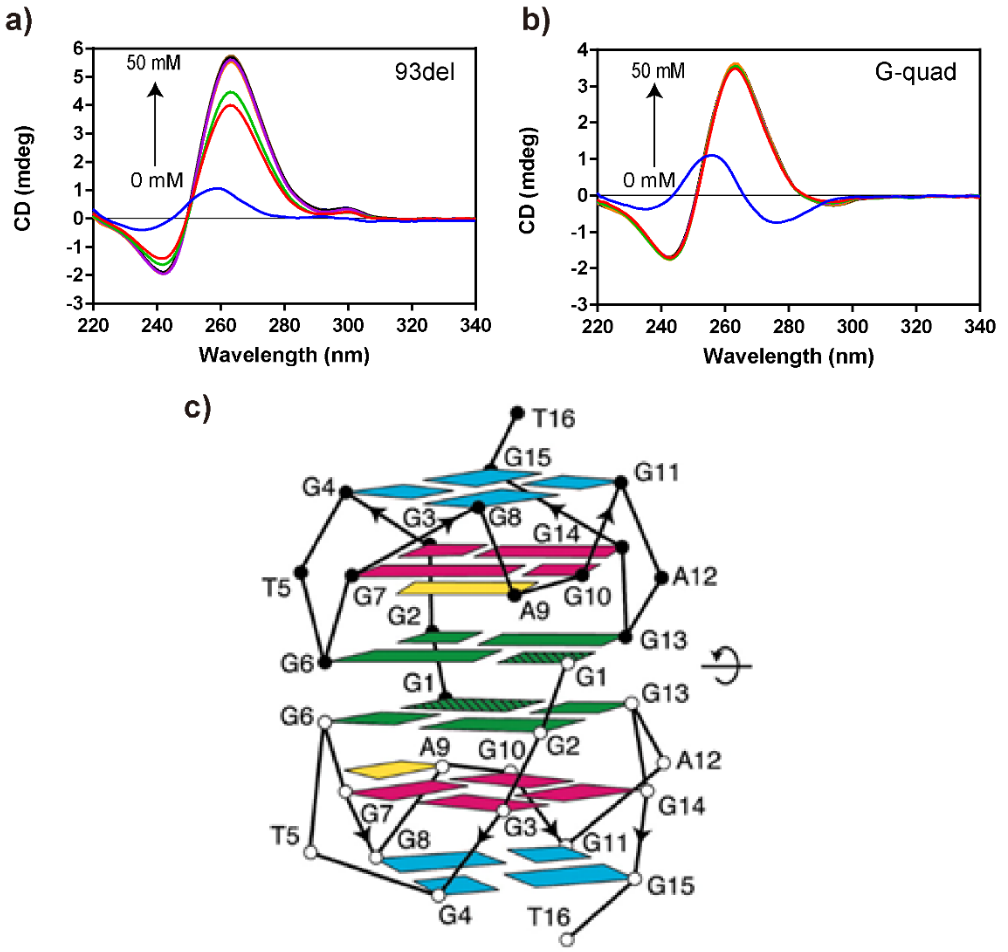

Figure S1. Optimization of candidate 1 (93del) and candidate 2 (G-quad) under different concentrations of KCl by circular dichroism (CD)[2,3]. (a) CD spectrum of 93del in 20 mM Tris-HCl; 93del was 5 μM. (b) CD spectrum of G-quad in 20 mM Tris-HCl; G-quad was 12.5 μM. Data are represented as mean ± SD (n = 3). The interval was set at 0.15 nm, and the spectral results were averaged from three scans that ranged from 220 to 340 nm. The background interference of Tris-HCl buffer solution was deducted from the CD data. (c) Schematic diagram of the dimeric quadruplex topology of 93del[2].

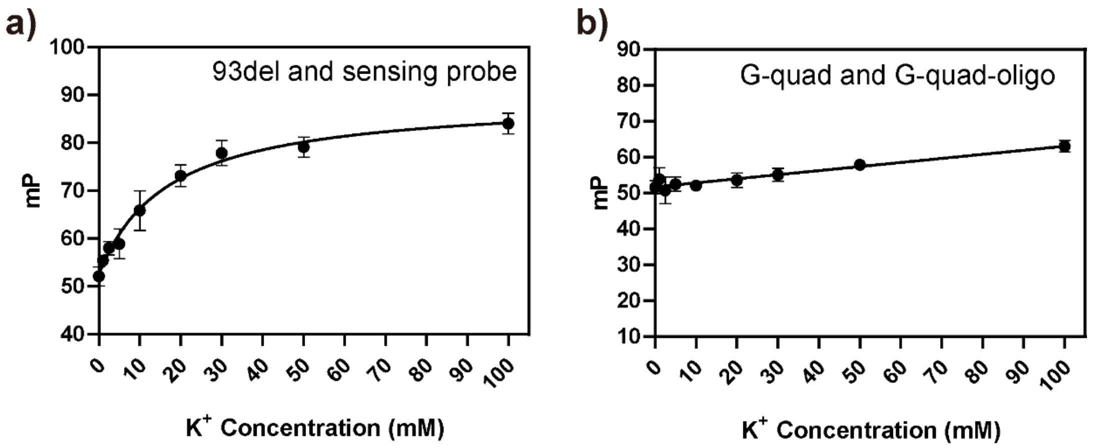

Figure S2. Optimization of candidate 1 (93del) and candidate 2 (G-quad) under different concentrations of KCl by fluorescence polarization. (a) FP data of 5'Alexa Fluor 488 labeled 93del and sensing probe in 20 mM Tris-HCl; 5'Alexa Fluor 488 labeled 93del and sensing probe were both 125 nM. (b) Fluorescence polarization analysis of 5'Alexa Fluor 488 labeled G-quad and G-quad-oligo in 20 mM Tris-HCl; 5'Alexa Fluor 488 labeled G-quad and G-quad-oligo were both 125 nM. The samples were incubated at room temperature for 30 minutes in 20 mM Tris-HCl containing varying concentrations of K^+ (0, 1.0, 2.5, 5.0, 10, 20, 30, 50, and 100 mM). Error bars show the standard deviation of three independent experiments.

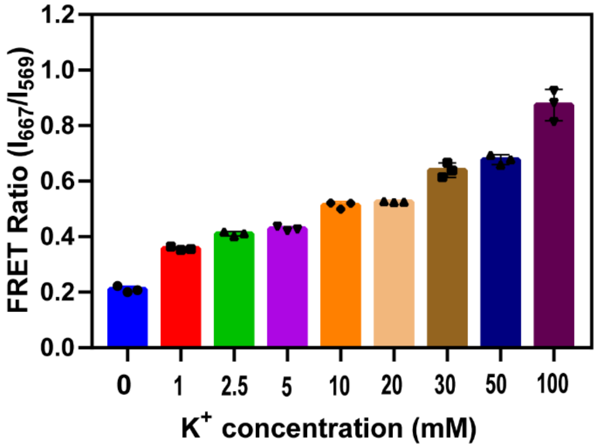

Figure S3. K^+-dependent FRET analysis. Different concentrations of K^+ (0, 1.0, 2.5, 5.0, 10, 20, 30, and 50 to 100 mM) were incubated in 20 mM Tris-HCl at room temperature for 30 min. FRET Ratio: Defined as the emission intensity of acceptor (Cy5, 667 nm) divided by that of donor (Cy3, 569 nm). Fluorescence measurements were carried out on a FluoroMax-4 spectrofluorometer using the kinetic analysis mode with excitation at 569 nm and emission at 667 nm, slit width, 2 nm.

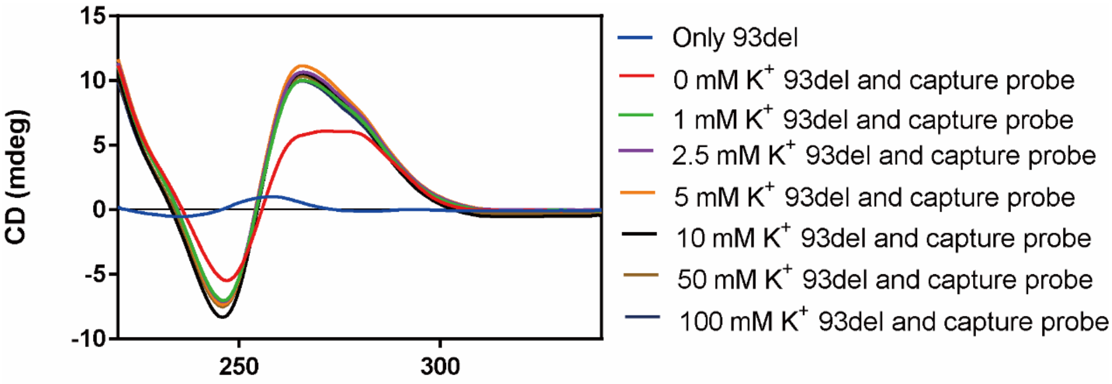

Figure S4. CD spectrum of 10 μM 93del and capture probe in 20 mM Tris-HCl under different concentrations of K^+, ranging from 0, 1.0, 2.5, 5.0, 10, 20, and 30 to 50 mM. The interval was set at 0.15 nm, and the spectral results were averaged from three scans, ranging from 220 to 340 nm. Background interference of the Tris-HCl buffer solution was subtracted from the CD data.

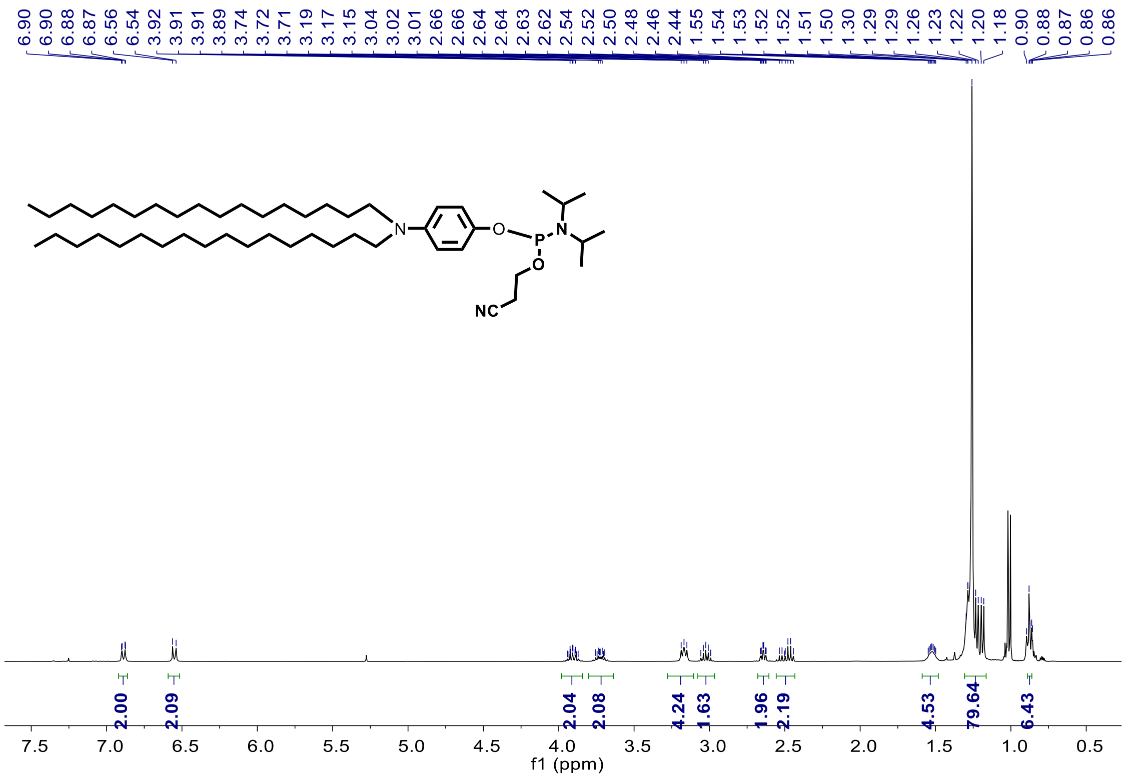

Figure S5. ^{1}H-NMR (400 MHz, $CDCl_3$) spectra of lipid phosphoramidite. ^{1}H NMR (400 MHz, $CDCl_3$) δ 6.89 (d, J = 9.0, 1.3 Hz, 2H), 6.55 (d, J = 9.1 Hz, 2H), 3.91 (dq, J = 8.3, 6.6 Hz, 2H), 3.73 (m, 2H), 3.18 (t, 4H), 2.66 (t, J = 6.6, 2.0 Hz, 2H), 1.53 (m, 4H), 1.18-1.36 (m, 72H), 0.88 (t, 6H).

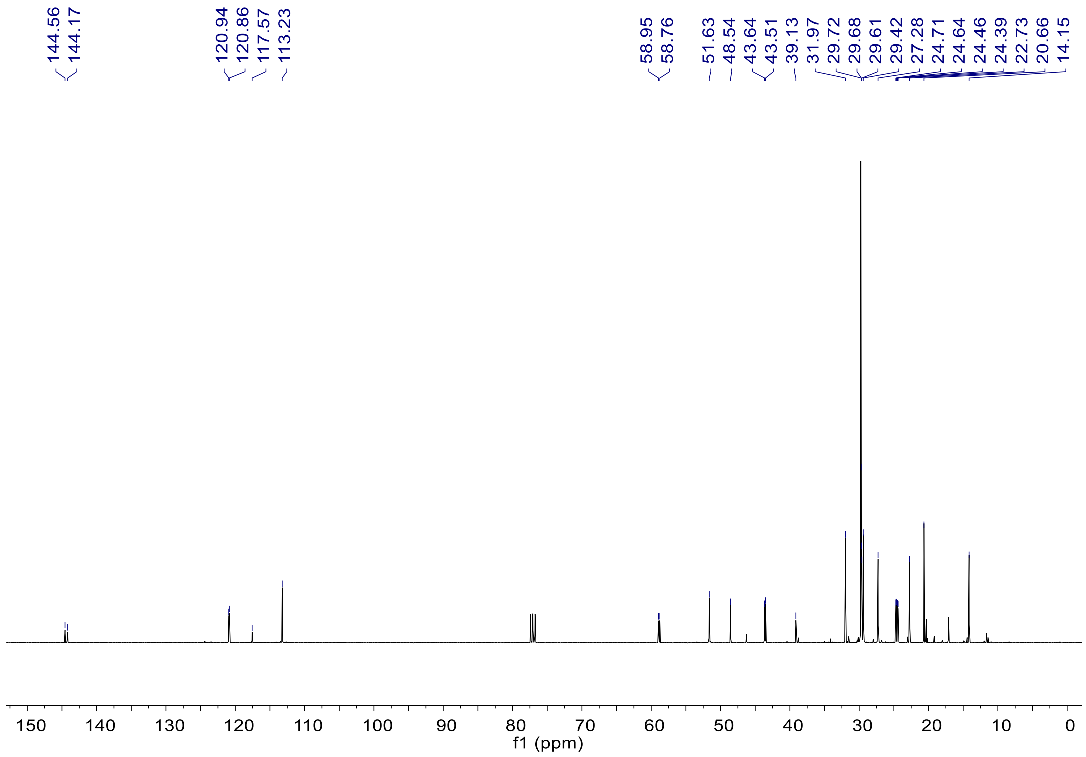

Figure S6. ^{13}C-NMR (100 MHz, $CDCl_3$) spectra of lipid phosphoramidite. ^{13}C NMR (100 MHz, $CDCl_3$) δ 144.56, 144.17, 120.94, 120.86, 117.57, 113.23, 58.95, 58.76, 51.63, 48.54, 43.64, 43.51, 39.13, 31.97, 29.72, 29.68, 29.61, 29.42, 27.28, 24.71, 24.64, 24.46, 24.39, 22.73, 20.66, 14.15.

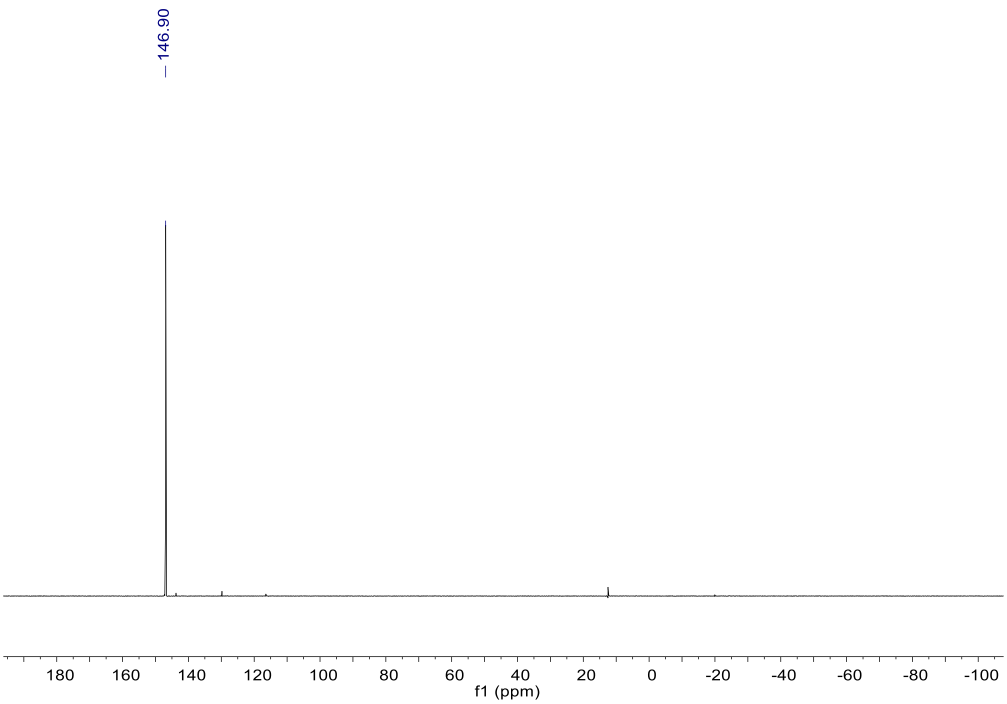

Figure S7. ^{31}P-NMR (162 MHz, $CDCl_3$) spectra of lipid phosphoramidite. ^{31}P NMR (162 MHz, $CDCl_3$) δ 146.90.

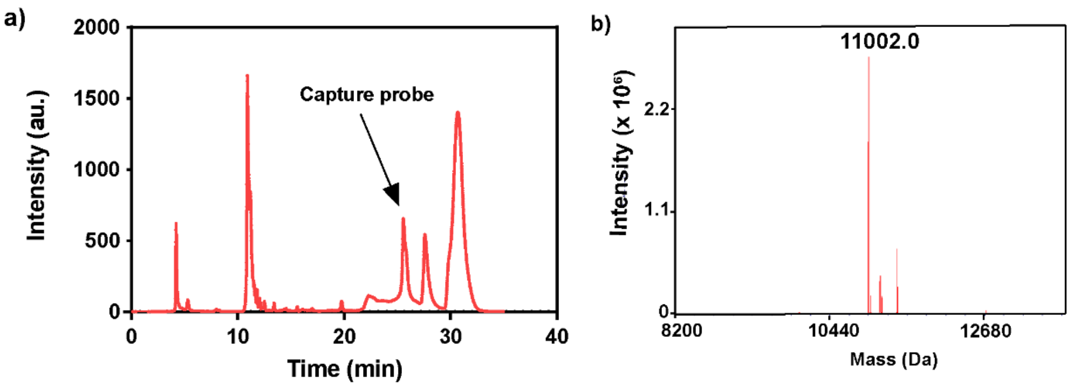

Figure S8. Purification and characterization of the capture probe. (a) HPLC purification of the capture probe. (b) Mass spectrometry analysis of lipid-labeled capture probe by Sangon (Shanghai). Calculated molecular weight: 11004.8 Da, Found: 11002.0 Da.

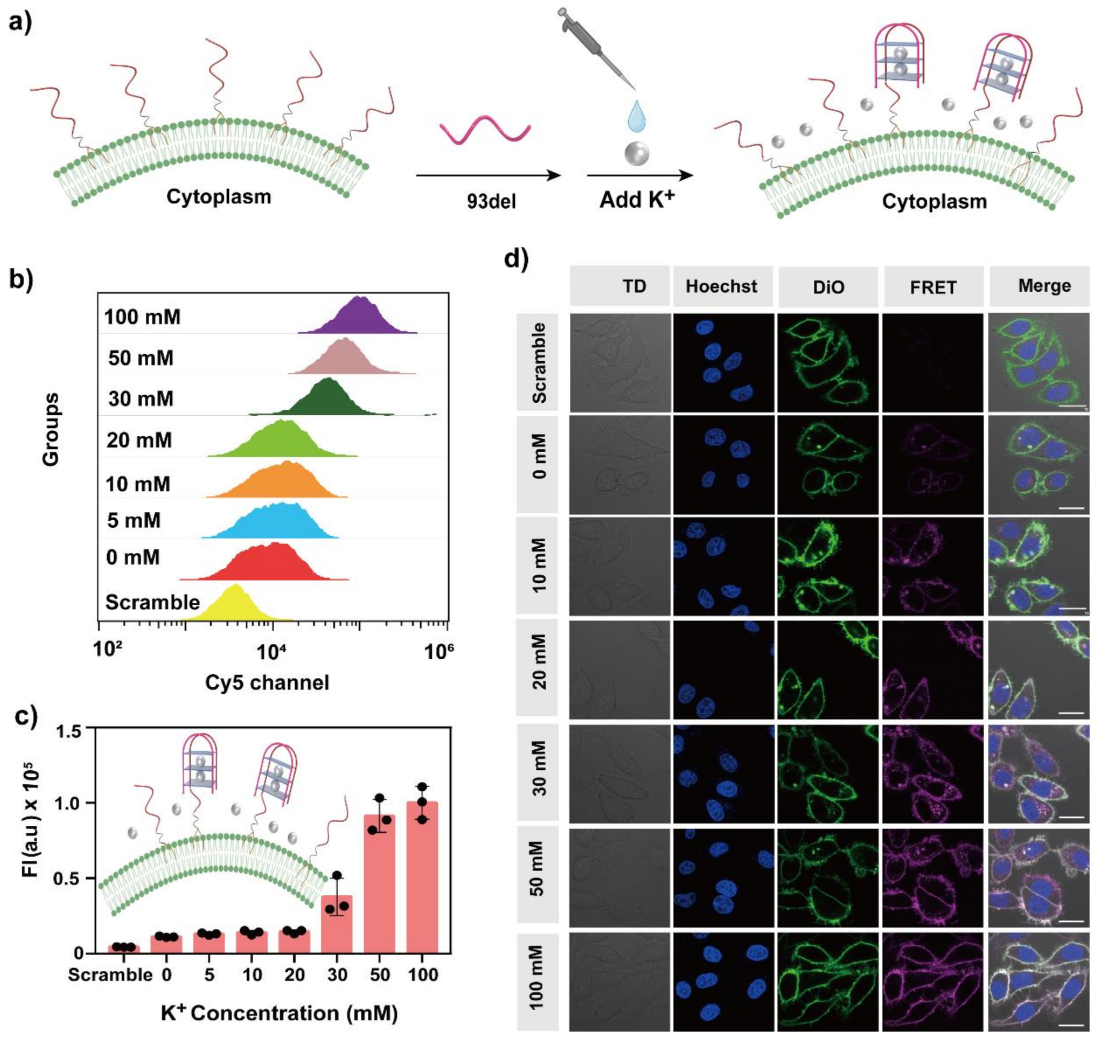

Figure S9. Fluorescence monitoring of K^+ with the split G4 Probe on the cell membrane. (a) Working principle of K^+ monitoring by capture probe with 93del. After the Cy3-labeled capture probe was anchored on the cell membrane, free Cy5-labeled 93del was captured in the presence of K^+ on the cell membrane. (b) Flow cytometry analysis of the formation of split G4 on the cell membrane in the presence of different K^+ concentrations, ranging from 0, 10, 20, 30, and 50 to 100 mM. (c) Statistics of fluorescence intensity corresponding to panel (b). All statistical data were collected from three independent experiments and presented as mean values ± SD. (d) CLSM imaging of Cy3-labeled 93del and Cy5-labeled 93del on the membrane of LoVo cells at different concentrations of K^+, ranging from 0, 10, 20, 30, and 50 to 100 mM. Scale bars, 20 μm.

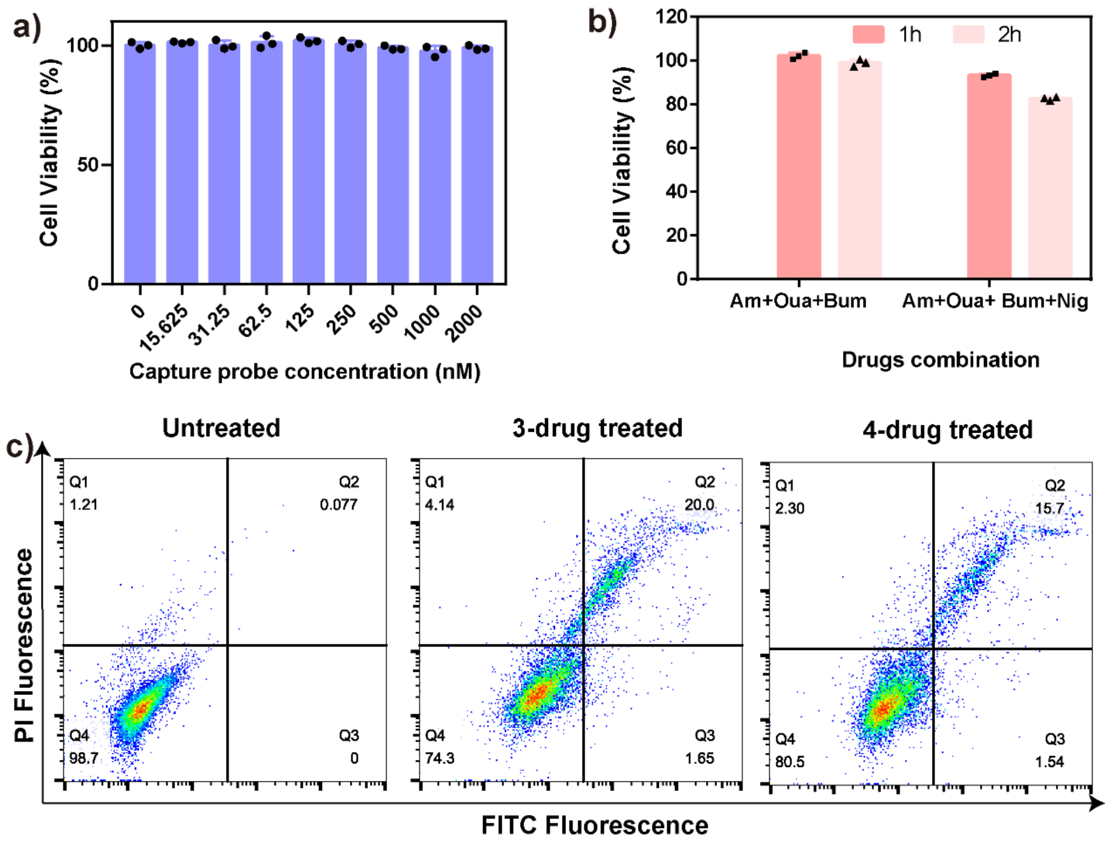

Figure S10. Cytotoxicity of capture probe and drug combinations. (a) Cytotoxicity of capture probe. Different concentrations of capture probe were incubated with LoVo cells for 2 h at 37°C, including 0, 15.625, 31.25, 62.5, 125, 250, 500, and 1,000 nM. (b) Cytotoxicity of drug combinations. The 3-drug or 4- drug group was incubated with LoVo cells for 1 h or 2 h at 37°C. Data are represented as mean ± SD. (n = 3). All experiments were conducted in triplicate, and relative cell viability (%) was expressed as a percentage in relation to untreated cells. (c) Apoptosis analysis by flow cytometry. Flow cytometry analysis of apoptosis in LoVo cells treated with combinations of drugs, apoptosis was assessed by Annexin V-FITC/PI dual staining after 2-hour treatment. Untreated cells (left), 3-drug combination (middle), and 4-drug combination (right) are shown.

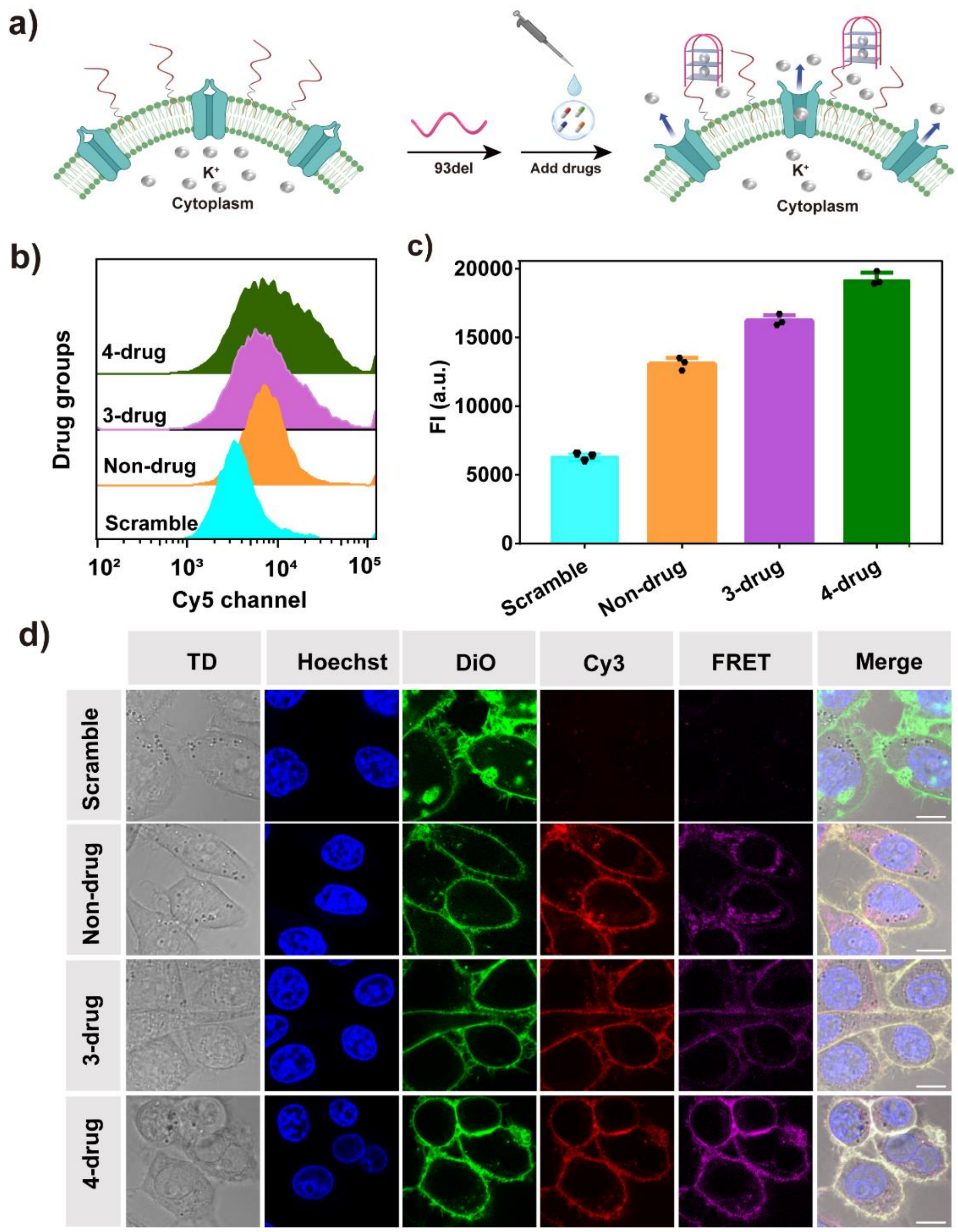

Figure S11. Drug-stimulated K^+ monitoring with the split G4 probe on the cell membrane. (a) Schematic illustration of formation of split G4 on the cell membrane by adding different drug combinations. (b) Flow cytometry analysis of the Cy5 signal for the 4-drug group, 3-drug group, and no drug group. (c) Quantification of the mean Cy5 fluorescence intensity from (b). All statistical data were collected from three independent experiments and presented as mean values ± SD. (d) CLSM imaging of Cy3-labeled 93del and Cy5-labeled 93del on the membrane of LoVo cells by the 4-drug group, 3-drug group, and no drug group treated. Scale bars, 10 μm.

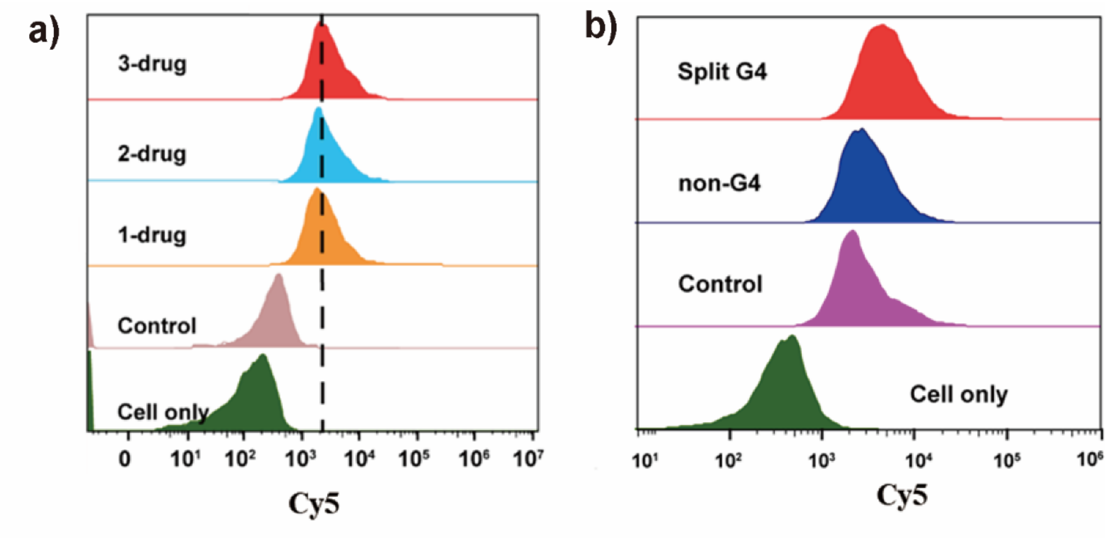

Figure S12. (a) Flow cytometry analysis showed the difference in Cy5 signal shift that resulted from treatment with the 1-drug group (Ampho), 2-drug group (Ampho B + Nig) and 3-drug group (Ampho B + Nig + Bum). (b) Flow cytometry analysis of G4 formation with specificity controls (The K^+ concentration is 10 mM). The Cy5 signal changes (split G4) reflect specific G4 formation, as demonstrated by comparison with non-G4-forming sequences.

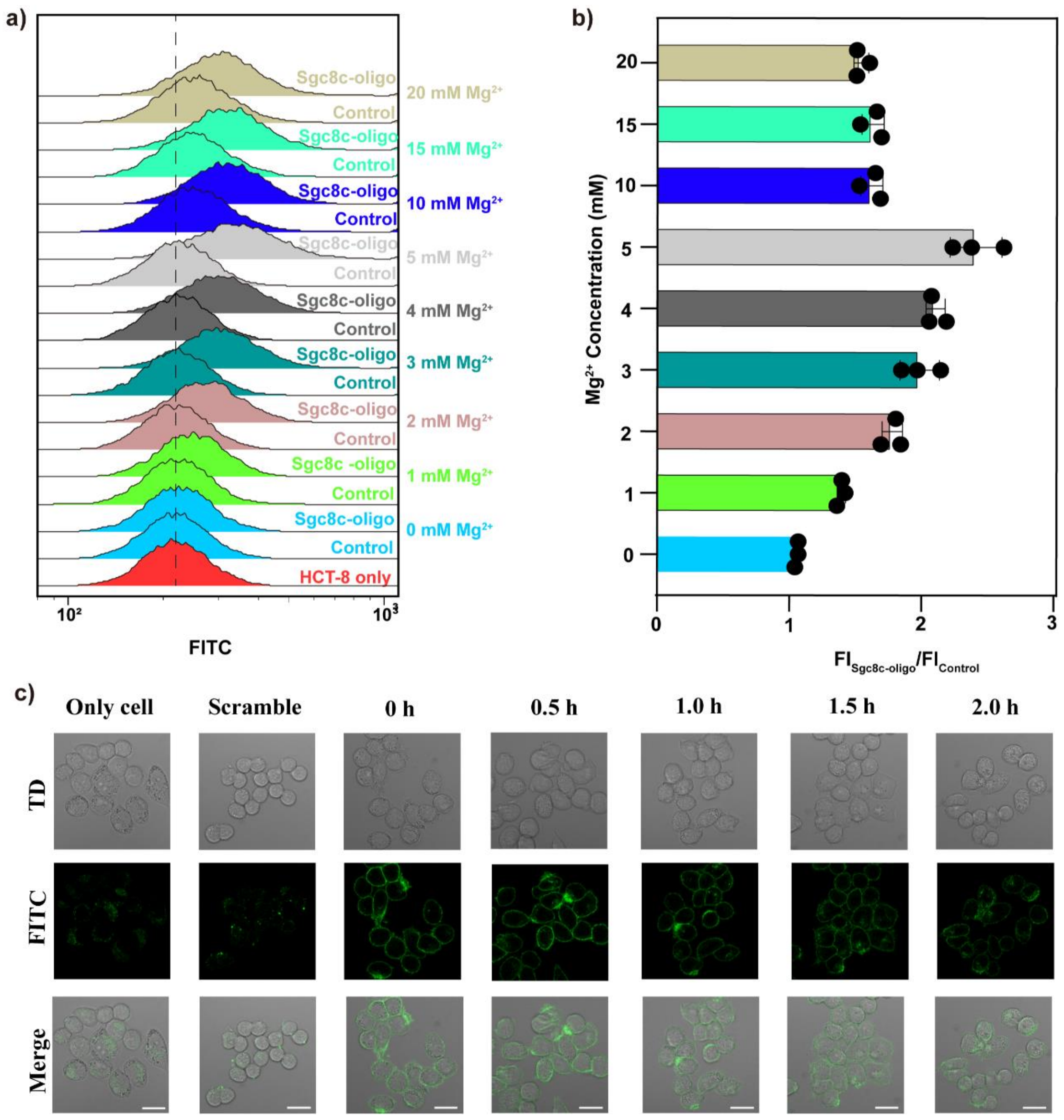

Figure S13. (a) The influence of Mg^{2+} on the binding affinity of the Sgc8c-oligo aptamer to HCT-8 cells was assessed by flow cytometry. Cells were incubated with the aptamer in binding buffers containing varying Mg^{2+} concentrations at 4 °C for 30 min. (b) Statistical analysis of fluorescence intensity corresponding to panel (a). All data are mean ± SD, n = 3. (c) Dynamic stability study of sgc8c-oligo binding to membrane protein PTK7. CLSM imaging (60x) of different time gradients in HCT-8 cells after incubating at 4 ℃ for 30 min. Scale bars, 20 μm.

a)

Groups	Cell numbers	Mean Aptamer number
Test1(1)	10000	2493
Test1(2)	10000	3623
Test2(1)	11684	247
Test2(2)	11772	244
Test3(1)	8454	271
Test3(2)	9580	211
Test4(1)	8644	95
Test4(2)	9047	62

b)

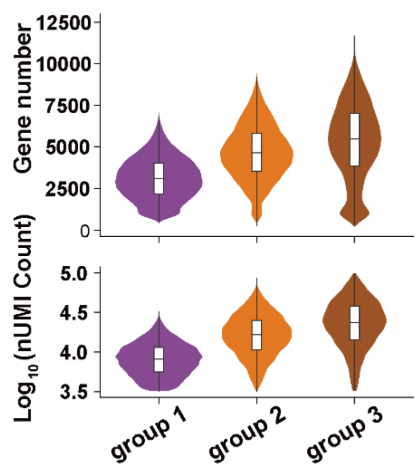

c)

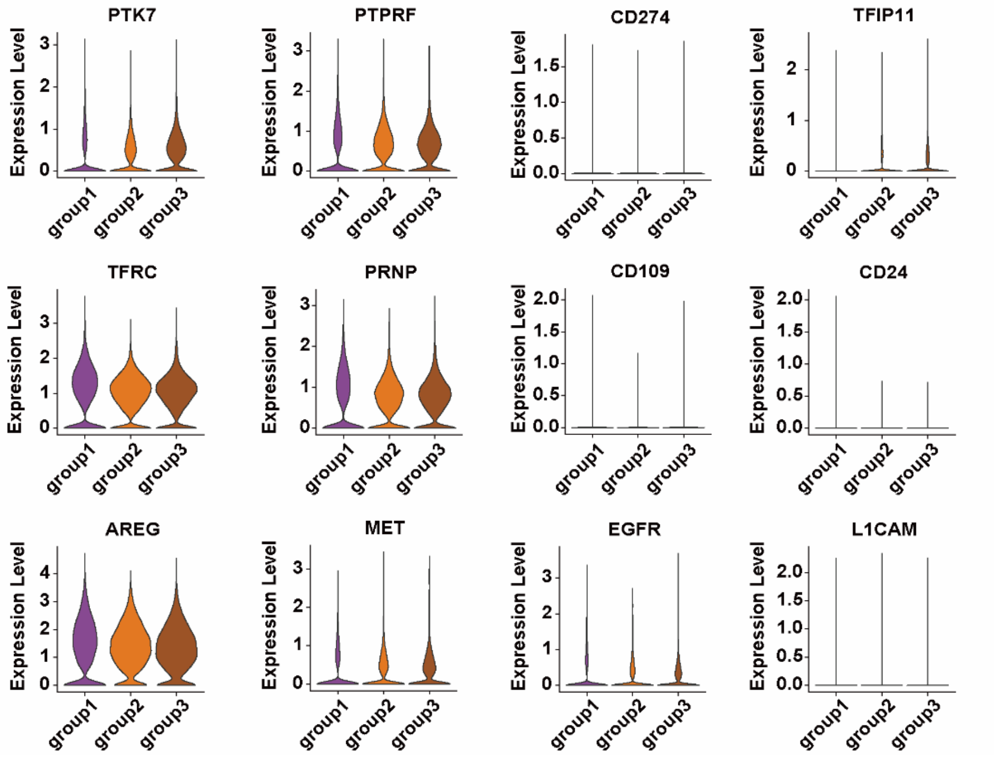

Figure S14. Optimization of aptamer capture efficiency under various conditions using the Ion-seq platform. (a) The table presented the mean number of aptamers captured under each condition. (b) Violin plot showed high-quality control over single-cell high-throughput sequencing in free oligonucleotide-treated(group1), scramble-treated(group2) and aptamer-treated groups(group3). (c) Violin plots showed that gene expressions of free oligonucleotide-treated, aptamer-treated and scramble-treated cells were comparable in both highly expressed genes and low-expressed genes.

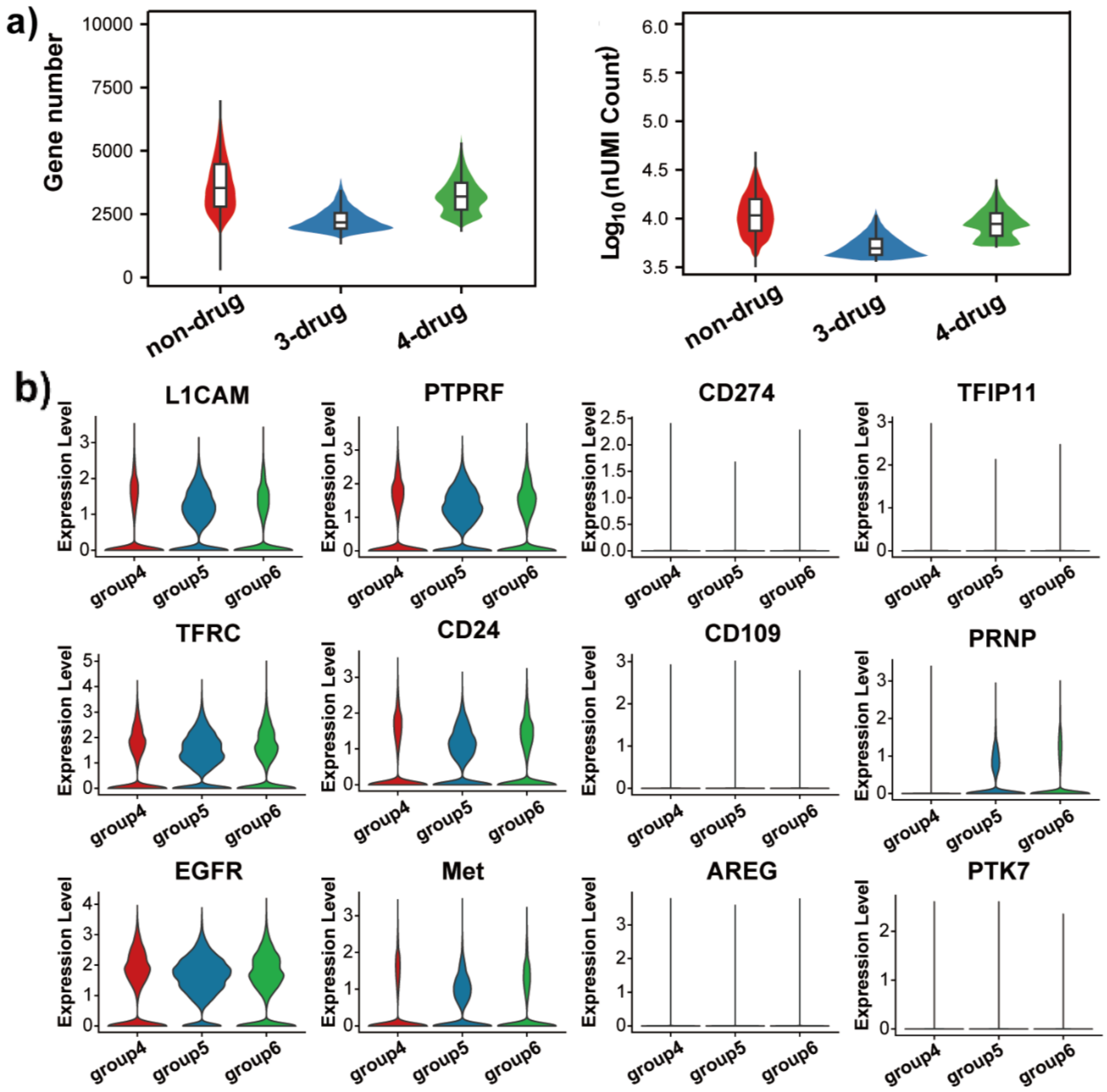

Figure S15. (a) Violin plot showed data quality control in group 4(non-drug treated), group 5(3-drug treated) and group 6(4-drug treated). (b) Violin plots showed both high- and low-expressing genes in group 4(non-drug treated), group 5(3-drug treated) and group 6(4-drug treated).

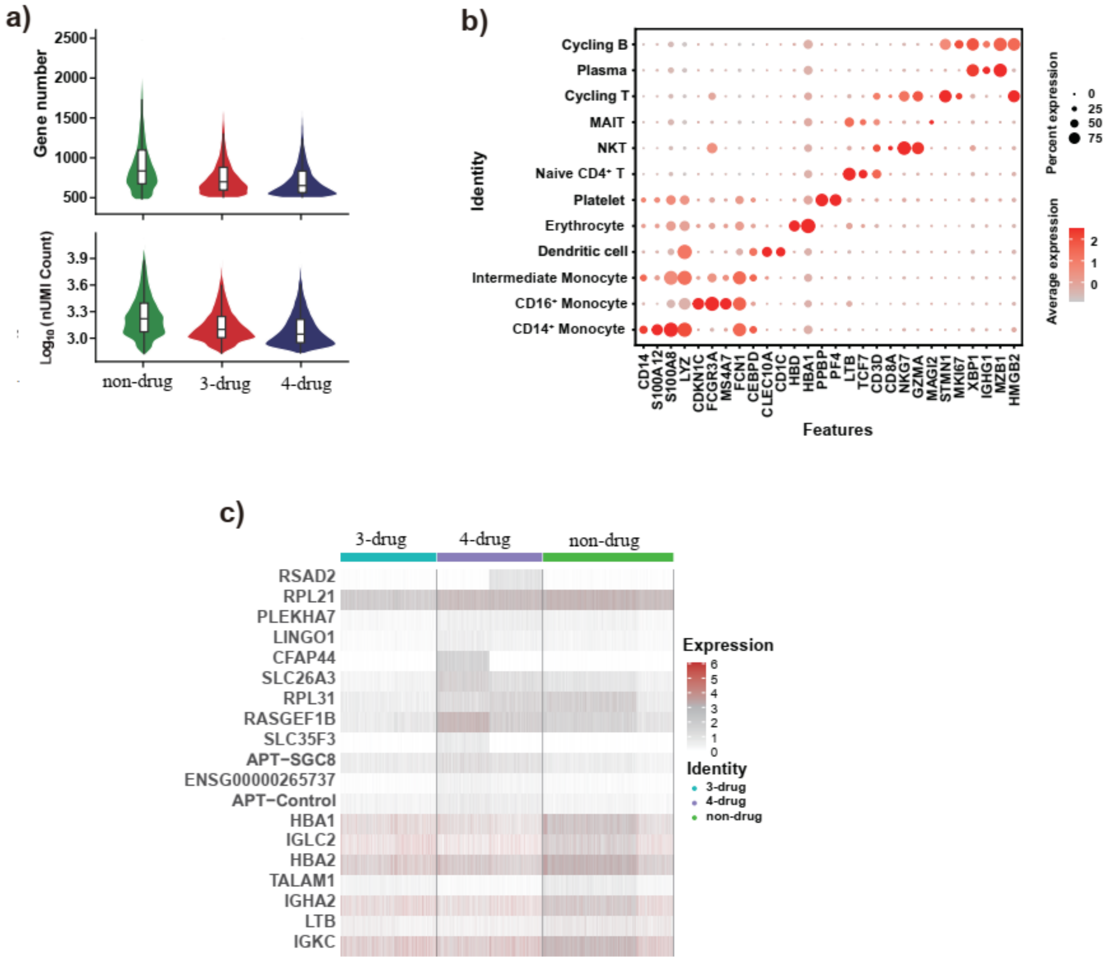

Figure S16. (a) Transcriptome datasets from 46,022 cells with an average of 1,000 genes and 2,500 UMI for each group (non-drug treated, 3-drug treated, and 4-drug treated). (b) Dot plots show marker gene expression in each cluster. (c) Heatmap show differentially gene expression in three groups (non-drug treated, 3-drug treated, and 4-drug treated).

7. Supplementary tables

Table S1. DNA sequences used in this study.

Name	DNA Sequence (5'-3')
93del	GGGGTGGGAGGAGGGT
Alexa Fluor 488-93del	Alexa Fluor 488-GGGGTGGGAGGAGGGT
Cy3-93del	Cy3-GGGGTGGGAGGAGGGT
Cy5-93del	Cy5-GGGGTGGGAGGAGGGT
Capture probe	Lipid-AAAAAAAAAAAAAAAGGGGTGGGAGGAGGGT
Cy3-capture probe	Lipid-GGGGTGGGAGGAGGGTAAAAAAAAAAAAAAA-Cy3
Sensing probe	TTGTCTTCCTAAGACCGCTTGGCCTCCGACTTaaaGGGGTGGGAGGAGGGTAAAAAAAAAAAAAAAAAAAAAAAA
Cy5-sensing probe	Cy5-TTGTCTTCCTAAGACCGCTTGGCCTCCGACTTaaaGGGGTGGGAGGAGGGTAAAAAAAAAAAAAAAAAAAAAAAA
G-quad	TGAGGGAGGGG
Alexa Fluor 488 -G-quad	Alexa Fluor 488-TGAGGGAGGGG
G-quad-oligo	TTGTCTTCCTAAGACCGCTTGGCCTCCGACTTaaaTGAGGGAGGGGAAAAAAAAAAAAAAAAAAAAAAAAA
Sgc8c	ATCTAACTGCTGCGCCGCCGGGAAAATACTGTACGGTTAGA
Sgc8c-oligo	TTGTCTTCCTAAGACCGCTTGGCCTCCGACTTaaaATCTAACTGCTGCGCCGCCGGGAAAATACTGTACGGTTAGAAAAAAAAAAAAAAAAAAAAAAAAAA
FITC-Sgc8c-oligo	FITC-TTGTCTTCCTAAGACCGCTTGGCCTCCGACTTaaaATCTAACTGCTGCGCCGCCGGGAAAATACTGTACGGTTAGAAAAAAAAAAAAAAAAAAAAAAAAAA
Scramble strand	TTGTCTTCCTAAGACCGCTTGGCCTCCGACTTaaaACCTAGCTGCTGCGGCTGCATACTGTACTGTCGAGAAATAGAAAAAAAAAAAAAAAAAAAAAAAAA
FITC-scramble strand	FITC-TTGTCTTCCTAAGACCGCTTGGCCTCCGACTTaaaACCTAGCTGCTGCGGCTGCATACTGTACTGTCGAGAAATAGAAAAAAAAAAAAAAAAAAAAAAAAA
Cy5- non-G4 strand	Cy5-NNNNNNNNNNNNNNNNNNNNNNNNNNNNN

The underlined letters represented the core sequence.

Table S2. HPLC program for purification of (Cy3-labeled) capture probe.

Time (min)	A (0.1 M TEAA)	B (Acetonitrile)
0	95%	5%
4	95%	5%
4.01	90%	5%
30	35%	65%
30.01	5%	95%
60	5%	95%

Table S3. Clinical samples form colorectal cancer patients.

No.	Gender	Age	Stage
P1	Male	44	CRC IV
P2	Male	48	CRC IV
P3	Male	68	CRC IV
P4	Male	69	CRC IV
P5	Female	52	CRC IV

8. Ethics statement

All methods were conducted according to relevant guidelines and regulations. Human serum samples were collected at Zhejiang Cancer Hospital. The study was approved by the Ethics Committee of Zhejiang Cancer Hospital and complied with all relevant ethical codes. All samples (n=5) were anonymous, and only gender, age, and pathological diagnosis were recorded. The ethical code is IRB-2024-1101 (IIT).

9. References

1. C. Jin, H. Zhang, J. Zou, et al. Floxuridine Homomeric Oligonucleotides "Hitchhike" with Albumin In Situ for Cancer Chemotherapy. *Angew, Chem, Int, Ed,* **2018,** *57*(29), 8994-8997.

2. T. Phan, V. Kuryavyi, B. Ma, A. Faure, et al. An interlocked dimeric parallel-stranded DNA quadruplex: a potent inhibitor of HIV-1 integrase. *Proc. Natl. Acad. Sci. U.S.A.* **2005,** *102*(3), 634-639.

3. L. Yang, Z. Qing, C. Liu, Q, et al., Direct Fluorescent Detection of Blood Potassium by Ion-Selective Formation of Intermolecular G-Quadruplex and Ligand Binding. *Anal. Chem.* **2016,** *88*(18), 9285-9292.

4. X. Guo, F. Chen, F. Gao, et al. CNSA: a data repository for archiving omics data. Database (Oxford). **2020**; 2020:baaa055. doi:10.1093/database/baaa055.

5. F. Chen, L. You, F. Yang, et al. CNGBdb: China National GeneBank DataBase, Hereditas. **2020**;42(08):799-809. doi:10.16288/j.yczz.20-080.
